# The fitness cost and benefit of phase‐separated protein deposits

**DOI:** 10.15252/msb.20178075

**Published:** 2019-04-08

**Authors:** Natalia Sanchez de Groot, Marc Torrent Burgas, Charles NJ Ravarani, Ala Trusina, Salvador Ventura, M Madan Babu

**Affiliations:** ^1^ Medical Research Council Laboratory of Molecular Biology Cambridge UK; ^2^ Bioinformatics and Genomics Programme Centre for Genomic Regulation (CRG) Barcelona Spain; ^3^ Universitat Pompeu Fabra (UPF) Barcelona Spain; ^4^ Systems Biology of Infection Lab Department of Biochemistry and Molecular Biology Universitat Autònoma de Barcelona Barcelona Spain; ^5^ Niels Bohr Institute University of Copenhagen Copenhagen Denmark; ^6^ Institut de Biotecnologia i Biomedicina and Departament de Bioquímica i Biologia Molecular Universitat Autònoma de Barcelona Barcelona Spain

**Keywords:** cell fitness, natural selection, phase separation, phenotypic diversity, protein deposit, Protein Biosynthesis & Quality Control, Quantitative Biology & Dynamical Systems

## Abstract

Phase separation of soluble proteins into insoluble deposits is associated with numerous diseases. However, protein deposits can also function as membrane‐less compartments for many cellular processes. What are the fitness costs and benefits of forming such deposits in different conditions? Using a model protein that phase‐separates into deposits, we distinguish and quantify the fitness contribution due to the loss or gain of protein function and deposit formation in yeast. The environmental condition and the cellular demand for the protein function emerge as key determinants of fitness. Protein deposit formation can influence cell‐to‐cell variation in free protein abundance between individuals of a cell population (i.e., gene expression noise). This results in variable manifestation of protein function and a continuous range of phenotypes in a cell population, favoring survival of some individuals in certain environments. Thus, protein deposit formation by phase separation might be a mechanism to sense protein concentration in cells and to generate phenotypic variability. The selectable phenotypic variability, previously described for prions, could be a general property of proteins that can form phase‐separated assemblies and may influence cell fitness.

## Introduction

The exposure of certain polypeptide segments in a protein to the solvent can trigger a process in which proteins phase‐separate into macromolecular assemblies (Veis, 2011). The formation of protein deposits can affect biological processes as a result of a loss and/or gain of function and has been implicated in disorders such as Alzheimer's or Parkinson's disease (Chiti & Dobson, 2006; Jahn & Radford, 2008; Babu *et al*, 2011; Gsponer & Babu, 2012; Sanchez de Groot *et al*, 2012; Ciryam *et al*, 2013; Lin *et al*, 2015; Woerner *et al*, 2016). However, protein sequences predisposed to form deposits are found in all kingdoms of life suggesting a neutral or advantageous effect on cell fitness (Li *et al*, 2012; Newby & Lindquist, 2013; Berchowitz *et al*, 2015; Khan *et al*, 2015; Chavali *et al*, 2017a). In fact, it has been shown that phase separation‐promoting sequences are essential to build membrane‐less structures and higher‐order assemblies with several biological functions (Maji *et al*, 2009; Bershtein *et al*, 2012; de Groot *et al*, 2012; Gsponer & Babu, 2012; Ciryam *et al*, 2013; Toretsky & Wright, 2014; Lin *et al*, 2015; Miller *et al*, 2015; Nott *et al*, 2015; Suresh *et al*, 2015; Wallace *et al*, 2015; Xiang *et al*, 2015; Zhu & Brangwynne, 2015; Chavali *et al*, 2017a; Holehouse & Pappu, 2018).

**Figure 1:**
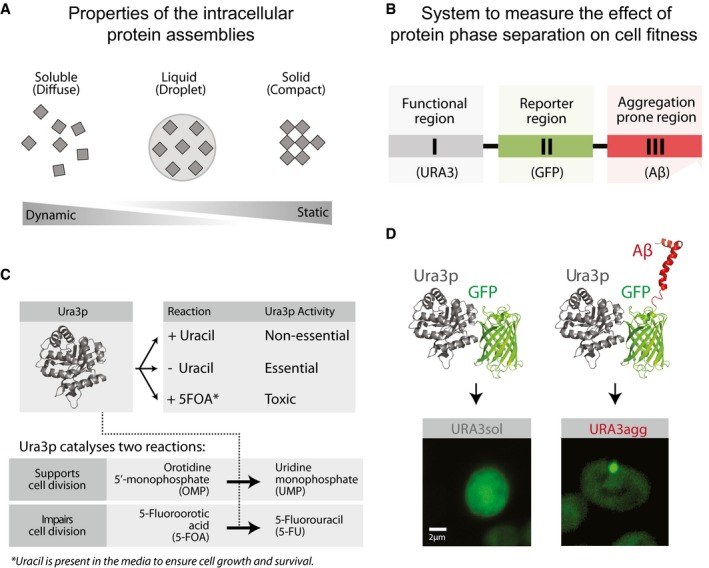
Model proteins and design of experimental system Protein phase separation can lead to the formation of protein assemblies with varying dynamics. Soluble proteins may phase‐separate into liquid‐like droplets or insoluble deposits that, according to their viscoelastic properties and their ability to exchange components with the cytoplasm, vary from being dynamic to static.Design of the modular system to measure the effect of protein phase separation on cell fitness.Growth media composition and the essential/non‐essential/toxic roles of Ura3p.The model proteins consist of a fusion between Ura3p and GFP (URA3_sol_, left) and the amyloid‐β‐peptide of 42 residues (Aβ) (URA3_agg_, right). After 18 h of expression, Ura3p_sol_ remains homogeneously distributed whereas Ura3p_agg_ is accumulated into intracellular foci.

Protein deposit formation due to phase separation inside the cell is a complex process that depends on a number of features such as the physicochemical properties of the polypeptide sequence (e.g., hydrophobicity, net charge; de Groot *et al*, 2006), local protein concentration (e.g., high vs. low concentration; Ciryam *et al*, 2013; Levy *et al*, 2014; Stepanenko *et al*, 2016), and its interaction with cellular components (e.g., chaperones, RNA; De Baets *et al*, 2011; Gsponer & Babu, 2012; Sanchez de Groot *et al*, 2012; Miller *et al*, 2015; Zhang *et al*, 2015; Jain *et al*, 2016; Pak *et al*, 2016; Maharana *et al*, 2018). The different combinations of these features result in the formation of deposits with different physicochemical and dynamic properties (Fig 1A). According to their viscoelastic characteristics and their ability to exchange components with the cytoplasm, protein assemblies can adopt a wide range of states that go from highly dynamic states with liquid droplet properties to almost static states with solid‐like properties (Fig 1A; Franzmann *et al*, 2018). In this manner, phase‐separated assemblies can be classified as liquid–liquid (e.g., nucleolus) or liquid–solid (e.g., amyloid aggregate) according to their viscoelastic and dynamic properties (e.g., deformation and coalescence; Morley *et al*, 2002; Jahn & Radford, 2008; Kaganovich *et al*, 2008; Escusa‐Toret *et al*, 2013; Toretsky & Wright, 2014; Lin *et al*, 2015; Miller *et al*, 2015; Zhang *et al*, 2015; Zhu & Brangwynne, 2015; Jain *et al*, 2016; Boeynaems *et al*, 2018; Franzmann *et al*, 2018; Alberti *et al*, 2019).

This variety of assemblies with distinct dynamic behavior (Fig 1A) is employed by cells to regulate key biological processes such as gametogenesis (Berchowitz *et al*, 2015), programmed necrosis (Li *et al*, 2012), hormone storage (Maji *et al*, 2009), or memory maintenance (Khan *et al*, 2015), among other functions (Toretsky & Wright, 2014; Miller *et al*, 2015; Chakrabortee *et al*, 2016; Mitrea & Kriwacki, 2016). Proteins that are able to phase‐separate can perform their biological functions in the soluble state such as the pituitary hormones (Maji *et al*, 2009); the phase‐separated state, such as the amyloid‐like form of Rim4 (Berchowitz *et al*, 2015); or in both states (e.g., monomeric Orb2 represses, whereas oligomeric Orb2 activates translation; Khan *et al*, 2015). Therefore, the formation of phase‐separated structures is not only associated with diseases but can positively influence cell fitness under different conditions. For instance, the recruitment of certain metabolic enzymes into cytoplasmic reservoirs/deposits enhances yeast survival during periods of starvation and stress (O'Connell *et al*, 2014; Petrovska *et al*, 2014; Suresh *et al*, 2015; Wallace *et al*, 2015; Riback *et al*, 2017; Franzmann *et al*, 2018). Thus, beyond prion domain‐containing proteins, the extensive number of reported self‐assembly events suggests that this is likely to be an intrinsic property of the polypeptide chain (Dobson, 1999; Monsellier *et al*, 2008). The ability for proteins to phase‐separate tends to be encoded in specific regions or domains (Appendix Fig S1; Moore *et al*, 2008; Marsh & Teichmann, 2010; Li *et al*, 2012; Berchowitz *et al*, 2015; Khan *et al*, 2015; Hervas *et al*, 2016). Such a modular organization permits independent evolution of protein regions and facilitates the emergence of proteins with new properties through recombination (Moore *et al*, 2008; Marsh & Teichmann, 2010).

The ultimate fitness consequences of phase‐separated structures will depend on the function of the protein, the components sequestered in the assembly, dynamic nature, and the molecular structure of the phase‐separated assembly, among other factors. Due to the complexity and the diverse effects associated with phase separation, there is a need to develop rational approaches that permit the quantification of the effects of forming such deposits on cell fitness in multiple environments and determine how proteins with phase separation‐prone segments are selected for, or against, in a cell population. To begin unraveling the effect of these factors, we have designed a model protein that phase‐separates to primarily form insoluble deposits and describe a population genetics approach. This allows us to disentangle and quantify the effect of the following factors associated with phase separation in different environments: the fitness change associated with (i) deposit formation, (ii) the loss of an essential biochemical activity of the protein that forms the deposit, and (iii) the gain of a beneficial effect due to deposit formation.

## Results

### Model for protein phase separation: one protein, three roles

**Figure EV1:**
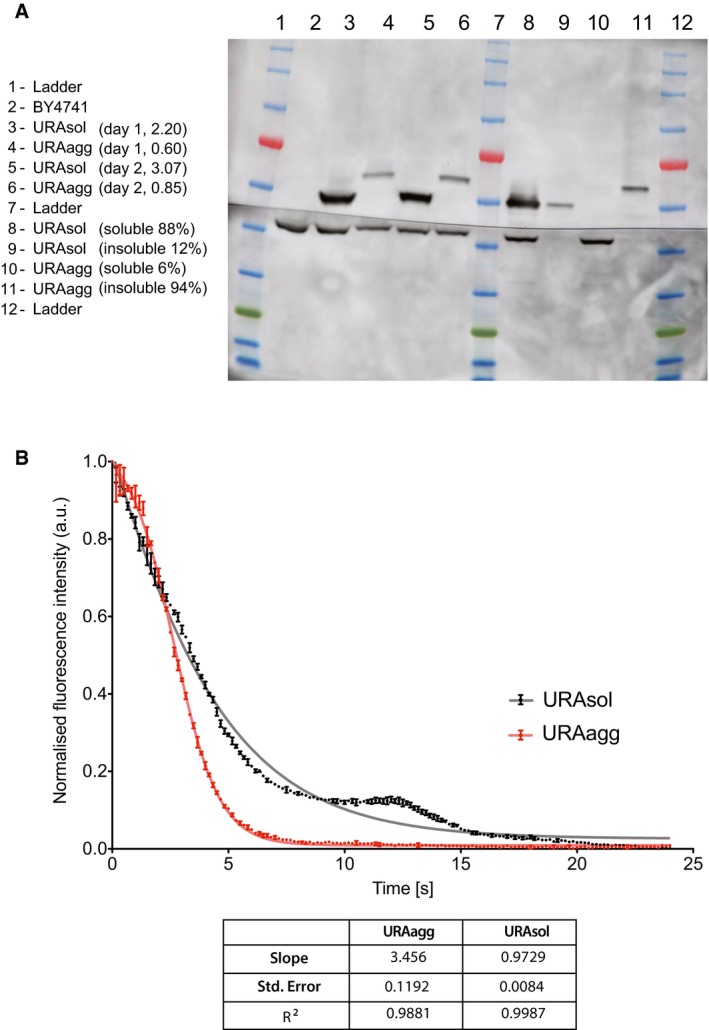
Protein levels and half‐life of URA3_sol_ and URA3_agg._ Analysis of the expression and the soluble/insoluble part of URA3_sol_ and URA3_agg_. Total protein fraction obtained by two independent experiments after 18 h of induction. The number indicates the Ura3p concentration (upper bands) quantification normalized by the concentration of PGK1p (lower bands). URA3_sol_ distribution is 88% soluble and 12% insoluble. URA3_agg_ is 6% soluble and 94% insoluble. Band analysis has been done with ImageJ.
*In vivo* degradation rate was measured as the fluorescence loss after induction inhibition (see the Materials and Methods section). The plot shows the fluorescence normalized by the concentration of cells (absorbance at 600 nm) at each time point. The slope (absolute value), standard error, and R^2^ of three technical replicates obtained from two different experiments are shown. The data were fitted to a Boltzmann's sigmoid.

Various studies have reported diverse and often conflicting effects in terms of the beneficial and detrimental effects of protein deposit formation on cell fitness (Maji *et al*, 2009; Geiler‐Samerotte *et al*, 2011; Sanchez de Groot *et al*, 2012; Escusa‐Toret *et al*, 2013; Tomala *et al*, 2014). These differences are understandable if one considers the complexity of deposit formation, the differences in the dynamic nature of the deposit, biochemical function of the protein forming the deposit, and the diversity in the experimental conditions in which the studies have been carried out. Furthermore, prior studies in the literature typically do not explicitly discriminate the different phase separation processes or the different fitness effects associated with proteins (e.g., loss vs. gain of function due to deposit formation). Here, we measure and disentangle the effects of phase separation of a model protein. To trigger the phase separation process, instead of mutating an endogenous protein to destabilize it as has been done before (Geiler‐Samerotte *et al*, 2011; Tomala *et al*, 2014), we designed a modular system that allows us to disentangle and quantify the cost/benefit of protein phase separation while tuning different roles influencing this process (e.g., the essentiality/non‐essentiality/toxicity of a protein). The model protein phase‐separates from a mainly soluble, functionally active state into a primarily insoluble, functionally less active state (Materials and Methods, Figs 5 and EV1, and Appendix Fig S2).

Mimicking the domain organization seen in nature (Derkatch *et al*, 1996; Moore *et al*, 2008; Marsh & Teichmann, 2010; Appendix Fig S1), our model protein is made up of three modular components (Fig 1B; Materials and Methods). The first component is a polypeptide segment whose biochemical activity can be essential, non‐essential, or toxic for the cell. We chose the endogenous yeast enzyme orotidine‐5′‐phosphate decarboxylase (Ura3p) involved in the production of pyrimidine nucleotides and widely employed for positive and negative selection (Seiple *et al*, 2006; Fig 1C). Ura3p activity is essential in yeast cells that are grown in the absence of uracil. However, Ura3p activity is non‐essential when grown in the presence of uracil. Furthermore, Ura3p activity is toxic in the presence of an alternative substrate named 5‐fluoroorotic acid (5FOA), as it leads to the production of a toxic compound (5‐fluorouracil, 5FU) leading to cell division arrest and cell death (Seiple *et al*, 2006; Fig 1C). The second component is a reporter (green fluorescent protein; GFP) that allows monitoring the integrity and the distribution/location of the protein in the cell (Fig 1B and D). The third component is a phase separation‐promoting segment that leads to the formation of intracellular, insoluble protein deposits, which in our system is the 42‐amino acid amyloid‐β‐peptide (Aβ) (de Groot & Ventura, 2006; Villar‐Pique & Ventura, 2013; Sanchez de Groot *et al*, 2015; Fig 1D).

We built two different constructs: one encoding Ura3p fused to GFP (to obtain a protein with no or low phase separation potential, Ura3p_sol_) and another that includes the Aβ peptide to promote the phase separation process (Ura3p_agg_) (Fig 1D; Materials and Methods). Instead of using Ura3p_sol_ as a control, we initially considered fusing Ura3p‐GFP to a soluble variant of the Aβ42 (Villar‐Pique & Ventura, 2013; Sanchez de Groot *et al*, 2015; e.g., a non‐foci‐forming variant). However, the different soluble variants are not always completely soluble in yeast, since after fractionation they are still found in the insoluble part and they form deposits in some of the stress environments investigated in the current work (Villar‐Pique & Ventura, 2013). Hence, we decided that the addition of a soluble variant of Aβ42 will not be a suitable control for generating the soluble version of Ura3p. We also decided against fusing another soluble protein with a length similar to Aβ42 but with a different sequence because URA3‐GFP fusion is already a large protein and the inclusion of a “random” short soluble peptide sequence might not affect the cost significantly.

**Figure EV2:**
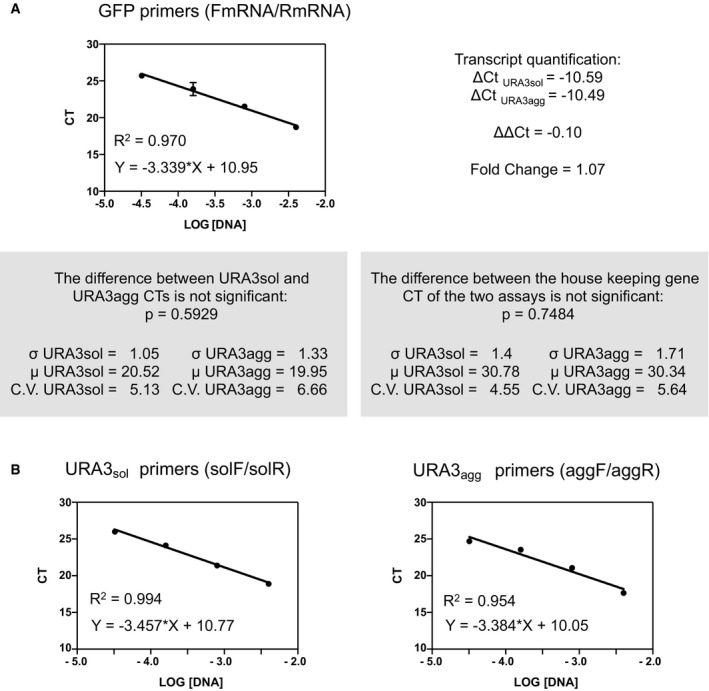
Variation in transcript expression and PCR efficiency URA3_sol_ and URA3_agg_ have similar mRNA abundance according to the ΔΔ*C*_t_ method (Teste *et al*, 2009). These measurements were made with a pair of primers that anneal to a common region located in the GFP sequence, named primers FmRNA and RmRNA, respectively (Materials and Methods). The standard curves obtained for these primers are shown.Standard curves obtained for the population quantification (solF/solR primers for URA3_sol_ cells and the aggF/aggR primers for URA3_agg_) (Materials and Methods).

**Figure EV3:**
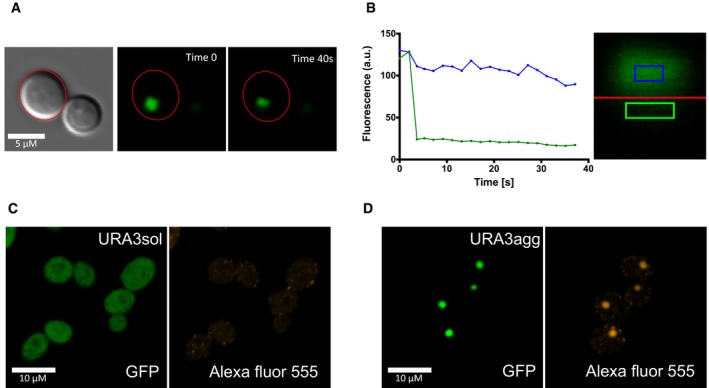
Properties of URA3_agg_ deposits using FRAP We tested the consistency of the URA3_agg_ foci by photobleaching one half of the deposit (green line and green square) and monitoring the protein diffusion from the other half (blue line and blue square) with confocal microscopy. The assay was monitored for 40 seconds and shows no fluorescence loss or gain in any of the sides. This indicates that these foci are very dense, very much like an insoluble protein deposit (IPOD; Kaganovich *et al*, 2008).
AFoci before (Time 0 s) and after (Time 40 s) photobleaching.BGraph showing the fluorescence changes along time in each side of the foci.C, DWe also tested the conformation of the protein enclosed in the URA3_agg_ foci by analyzing the binding of anti‐oligomer antibodies (rabbit anti‐oligomer (A11) AHB0052). As a secondary antibody, we used goat anti‐rabbit IgG H&L tagged with an Alexa Fluor® 555 (ab150078). We obtained a bright fluorescence signal that colocalizes with the foci's GFP fluorescence, indicating that the deposits are rich in oligomeric structures. As a control, we also incubated URA3_sol_ with these antibodies obtaining few faint Alexa Fluor foci at the cytoplasm, indicating the absence or very low presence of oligomers.

We integrated the two chimeric genes (Ura3p_sol_ and Ura3p_agg_) into a stable genomic region (TRP1 locus) to ensure steady expression in multiple generations. We included an inducible promoter (GAL1) to control transcription and guarantee expression under different environments (Materials and Methods; Appendix Fig S2). Their integration in the *S. cerevisiae* genome resulted in two strains (URA3_sol_ and URA3_agg_) with the same genomic background and with similar mRNA expression levels (Fig EV2). This ensures that the fitness cost of expressing the constructs as gratuitous proteins will remain similar in the two strains (Dekel & Alon, 2005; Pena *et al*, 2010; Plata *et al*, 2010; Geiler‐Samerotte *et al*, 2011; Kafri *et al*, 2016) and hence minimally influence the measurements of fitness effects (next section). We monitored competitive growth and formation of deposits in different environments by changing the osmotic pressure (1 M sorbitol, 0.5 M NaCl), oxidation level (0.5 mM H_2_O_2_, 1 mM DTT), and temperature (37, 30 and 25°C) and in the presence of a chemical chaperone (0.5 M proline). After 18 h of induction at standard growth conditions (30°C) in mid‐log phase, Ura3p_sol_ remains distributed through the cytoplasm, whereas Ura3p_agg_ forms stable, non‐dynamic protein deposits similar to IPODs (insoluble protein deposits; Kaganovich *et al*, 2008), as measured by FRAP experiments (Figs 1D and EV3). Although some of these assemblies may contain other proteins (Rothe *et al*, 2018), prior findings suggest that the vast majority of them are likely to be Ura3p_agg_ (Morell *et al*, 2011; Sanchez de Groot *et al*, 2015).

### Quantifying selection for/against phase separation in different environments

**Figure 2:**
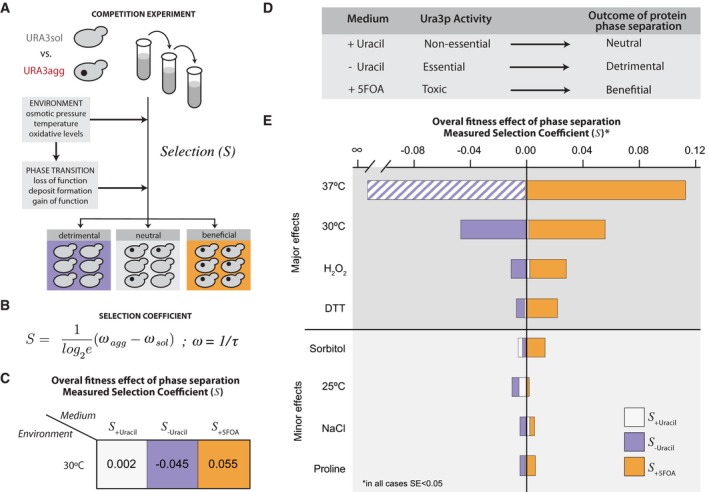
Protein phase separation and selection coefficient AExperimental design to measure the selection coefficient (*S*) upon growing URA3_agg_ in competition with URA3_sol_.BThe selection coefficient, *S*, is proportional to the difference in growth rates between URA3_agg_ and URA3_sol_ (Materials and Methods). ω is the growth rate, which corresponds to the inverse of doubling time, τ.C, DThe *S*‐values measured at standard conditions (30°C) indicate that depending on the media composition (+Ura, −Ura, +5FOA), the formation of protein deposits can be neutral, deleterious, or even advantageous for the cell as the function becomes non‐essential, essential or toxic, respectively.E
*S*‐values measured in different environments. In all cases, the standard error is below 5%. See also Appendix Table S1. Color scale: purple in the absence of uracil (‐Ura), white in the presence of uracil (+Ura), and orange in the presence of 5FOA (+5FOA). At 37°C, the heat stress together with the inability to fold Ura3p has such a strong fitness effect that the strains barely grew in the absence of uracil, impeding the measurement of *S*. A bar of purple‐white diagonal lines indicates a presumed value of *S* at 37°C without uracil.

We experimentally determined how the formation of intracellular protein deposits is selected for, or against, in a population when the two strains (URA3_sol_ and URA3_agg_) are grown in competition (Fig 2A). We grew mixed cell cultures (1:1 initial proportion) for three days in exponential phase and measured the selection coefficient (*S*) by PCR (Fig 2A; Appendix Table S1; Materials and Methods; Chevin, 2011; Geiler‐Samerotte *et al*, 2011; Sanchez de Groot *et al*, 2015). This growth phase will ensure a constant doubling time, a young population, and a low number of aged protein assemblies (Hill *et al*, 2016). The selection coefficient (*S*) is related to the difference in growth rate between the two strains (Fig 2B) and quantifies how much the cell fitness increases (positive values) or decreases (negative values) due to the formation of deposits in URA3_agg_ in comparison with URA3_sol_.

**Figure EV4:**
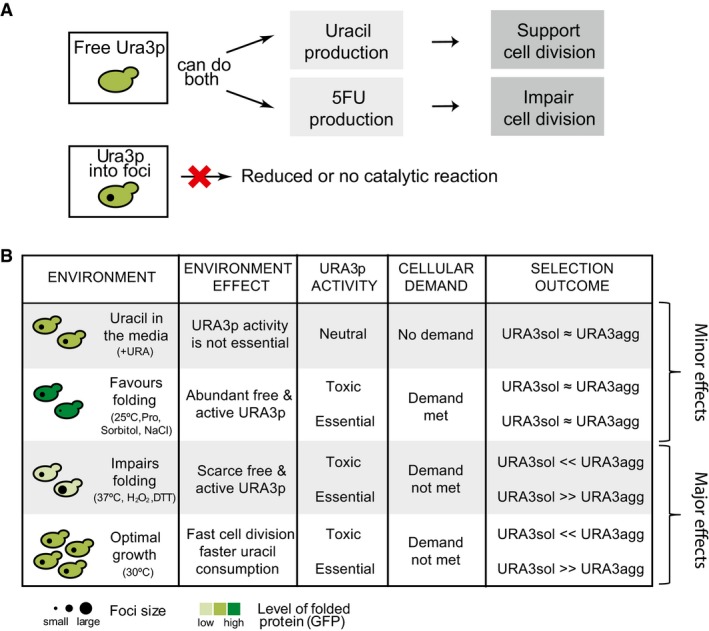
Summary of the effects associated with URA3_agg_ phase separation in different environments that affect the topology of Ura3p, and hence protein activity (supply) as well as the cell state (demand) Diagram showing different activity associated with the free and deposited protein.Summary table of the effects associated with Ura3p_agg_ deposition and their effects on the selection outcome when growing in competition with URA3_sol_.

The results obtained at standard growth conditions (30°C) show that the formation of Ura3p_agg_ deposits can be neutral (in the presence of uracil; non‐essential), deleterious (in the absence of uracil; essential), or even advantageous (in the presence of 5FOA; toxic) for yeast, depending on the composition of the growth medium (Fig 2C; average *S*‐values from two biological replicates). Therefore, without changing the protein sequence or the genotype, we can quantify different overall effects of deposit formation on cell fitness (Fig 2D). In this manner, the system we developed allows for the quantification of the overall effects (both negative and positive fitness effects) of deposit formation upon protein phase separation in different environments (Fig 2E and Appendix Table S1). We find a wide range of selection coefficients suggesting that URA3_agg_ is differentially selected for or against when the role of the protein is different (essential/non‐essential/toxic; in different media) and in different environments (Fig 2E). Changing the oxidation levels (0.5 mM H_2_O_2_, 1 mM DTT) or increasing the temperature (37°C) has a major effect on fitness compared to conditions in which we changed the osmotic pressure (1 M sorbitol, 0.5 M NaCl), or decreased the temperature (25°C) or when we added a chemical chaperone (0.5 M proline). This observation raises the question as to how and why the phase separation of the same protein leads to such differences in selection coefficients under different environments. We investigate this question in the next sections (Fig EV4).

### Disentangling the different effects of protein phase separation

**Figure 3:**
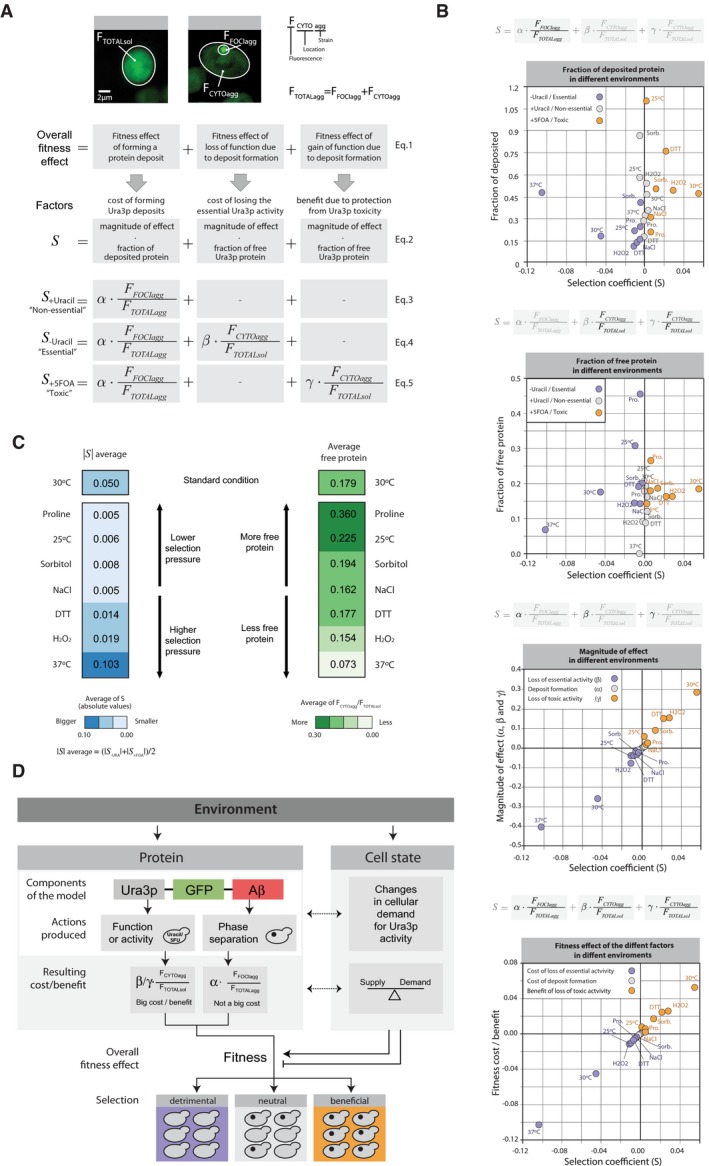
Fitness cost and benefit analysis We measured the intensity of fluorescence emitted by the fluorescent reporter (GFP) to estimate (i) the amount, (ii) the location, and (iii) the deposited/diffused state of the model proteins. Accordingly, for each cell analyzed, we measured the fluorescence intensity of its cytoplasm (*F*_*TOTALsol*_ for URA3_sol_ and *F*_*CYTOagg*_ for URA3_agg_) and of its foci (*F*_*FOCIagg*_). We used these measurements to estimate the different effects of protein phase separation on cell fitness. The overall fitness effect of forming a protein deposit depends on three factors: foci formation, loss of function, and gain of function (Equation (1)). In our system, we can distinguish between the cost of forming Ura3p deposits, cost of losing the essential Ura3p activity, and the benefit from protection against Ura3p toxicity. Each cost and benefit effect depends on the ratio of deposited or free protein and the specific environment. Accordingly, we can split these cost and benefit effects into two components: one associated with the amount of deposited or free protein (*F*_*FOCIagg*_ */F*_*TOTALagg*_ or *F*_*CYTOagg*_ */F*_*TOTALsol*_) and a magnitude of effect defined by the environmental conditions (α, β, or γ) (Equation (2)). For the non‐essential, essential, and toxic roles (+Ura, −Ura, and +5FOA) of Ura3p, the number and type of effects applicable are different (equations (3), (4), (5)).Plots showing the distribution and relationship between the fractions of deposited or free Ura3p, its magnitude of effect on fitness (α/β/γ), and the selection coefficient (*S*) in different environments.Average of the selection coefficient (left) and average of the cytosolic fluorescence (equivalent to the fractional abundance of the free protein in URA3_agg_, right).Schematic representation highlighting how phase separation of the same protein can result in different fitness effects depending on the environment.

The overall fitness effect (cost/benefit) of protein phase separation is not only determined by the loss or gain of the biochemical activity of the protein, but also due to the cost of deposit formation (e.g., amino acid sequestration in deposits, sequestration of ATP‐dependent chaperone activity and other proteins, toxicity of the assembly; Maji *et al*, 2009; Olzscha *et al*, 2011;; Gsponer & Babu, 2012; Sanchez de Groot *et al*, 2012; Suraweera *et al*, 2012; Tomala *et al*, 2014; Patel *et al*, 2017); Fig 3A, equation (1)). To infer the impact of these effects, we considered that the measured selection coefficient (*S*) is determined by a combination of three factors: (i) the cost of deposit formation, (ii) the cost of losing the essential Ura3p biochemical activity, and (iii) the benefit of gaining a protective function against the toxic activity by sequestering Ura3p into deposits (Fig 3A, equation (2)). In conditions where Ura3p is not essential (with uracil), any change in cell fitness primarily depends on the fitness cost of deposit formation (Fig 3A, equation(3)). In the absence of uracil, the overall effect on fitness includes not only the cost of deposit formation but also the cost of reducing Ura3p activity due to deposit formation (Fig 3, equation (4)). Finally, in the presence of 5FOA, the effect on fitness includes the cost of deposit formation, and the fitness benefit of reducing the toxic activity of Ura3p by sequestering the protein into the deposit (Fig 3, equation (5)). Although some Ura3p activity can be present in deposits (O'Connell *et al*, 2014; Wallace *et al*, 2015), this is likely to be significantly reduced (e.g., due to protein conformational changes, and restricted access to substrate) compared to the free, diffusible well‐folded protein as in the URA3_sol_ strain (Suresh *et al*, 2015).

To relate the measured selection coefficient with the different effects of deposit formation, we quantified the total fluorescence (F_TOTALagg_) and the amount of fluorescence in deposit/foci (F_FOCIagg_), and the cytosol (F_CYTOagg_) for individual cells of the URA3_agg_ population using confocal microscopy (Fig 3A). The measured fluorescence is proportional to the amount of protein present in the cell (Soboleski *et al*, 2005). For the URA3_sol_ strain, the total fluorescence (F_TOTALsol_) is the same as cytosolic fluorescence (F_CYTOsol_) and is indicative of the maximum amount of free, active Ura3p that can be available in our designed system (Materials and Methods). The cost of forming a deposit is defined as the product of the fraction of the protein in deposits (phase‐separated) (F_FOCIagg_/F_TOTALagg_) and a proportionality constant/coefficient (α) that depends on the environment (Fig 3A, equations (2) and (3)). This constant/coefficient is indicative of the magnitude of the effect of forming the protein deposit on the fitness in a particular environment and is considered to remain the same for an environment irrespective of whether Ura3p is non‐essential, essential or toxic (i.e., when grown in +Uracil, −Uracil or +5FOA; see Materials and Methods). The effect of reduced Ura3p activity due to deposit formation is defined as the product of the apparent free, active protein (F_CYTOagg_/F_TOTALsol_, ratio of free protein in the URA3_agg_ strain to the maximum that can be available in a cell) and a proportionality constant/coefficient (β for cost/γ for benefit) that is indicative of the magnitude of the effect of the loss/gain of biochemical activity on fitness in a particular environment (Fig 3A, equations (4) and (5), Materials and Methods). Although both strains (URA3_agg_ and URA3_sol_) present similar transcript levels (Fig EV2), due to deposit formation, a fraction of translated Ura3p_agg_ can be rapidly removed through autophagy or other mechanisms (Villar‐Pique & Ventura, 2013; Sanchez de Groot *et al*, 2015; Fig EV1). This may hence lead to a reduction in F_TOTALagg_ when compared to F_TOTALsol_ (Villar‐Pique & Ventura, 2013; Sanchez de Groot *et al*, 2015; Fig EV1; Appendix Fig S2; Appendix Table S1; Materials and Methods).

### Environment modulates the magnitude of the effects of phase separation on cell fitness

Environment can modulate the selection pressure on variants in a population; genotypes and characteristics that are beneficial in certain environments could become detrimental in others (Pena *et al*, 2010; Bershtein *et al*, 2012). We examined how the fraction of deposited vs. free Ura3p, the magnitude of effect, the fitness cost/benefit of deposit formation, and the selection coefficient (*S*) vary in different environments.

When Ura3p activity is not essential for a cell, although the fraction of the deposited protein varies considerably in the different environments, the measured selection coefficient remains closer to 0 (0 means no difference in cell fitness; Fig 3B, first plot; gray dots). However, when Ura3p activity is essential or toxic, the fraction of the deposited protein in the different environments and the selection coefficient tend to vary considerably (e.g., *S *=* *−0.103, without uracil at 37°C and *S *=* *0.055, with 5FOA at 30°C) (Fig 3B, first plot; purple and orange dots). Consistently, in terms of the fraction of free Ura3p, although the amount of free protein varies in the different environments, the measured selection coefficient does not differ much when Ura3p is not essential (Fig 3B, second plot; gray dots). When Ura3p is essential or toxic, the fraction of free protein in the different environments and the selection coefficient tend to vary considerably (Fig 3B, second plot; purple and orange dots). In addition, we find that the magnitude of effect for the cost of deposit formation (α) is considerably lower than that of the effects associated with protein function, both with loss of essential enzymatic activity (β) and gain of protection against the toxic activity (γ) in the different environments (Fig 3B, third plot). These observations quantify and reflect the essentiality and lethality associated with the biochemical reaction catalyzed by Ura3p. Moreover, whereas the cost of deposit formation remains relatively stable in the different environments (Fig 3B, fourth plot; gray dots), the fitness cost/benefit associated with reduced Ura3p activity varies significantly in different environments. Thus, in certain environments the amount of free Ura3p is more important for cell fitness than in others (e.g., 37°C and 25°C, without uracil; Fig 3B, second plot). In line with this observation, the environments with higher average absolute selection coefficient (Fig 3C, darker blue) tend to have lower average apparent free protein in the URA3_agg_ strain (Fig 3C, lighter green; Pearson correlation coefficient = −0.64).

Due to the modular nature of the designed protein, a change in the environment can (i) affect the phase separation process (e.g., higher temperature can accelerate deposit formation; de Groot & Ventura, 2006) and/or (ii) affect the activity of Ura3p, for instance, by enhancing protein folding (e.g., presence of the chemical chaperone proline; De Los Rios & Goloubinoff, 2012) or misfolding (e.g., higher levels of oxidative stress). Interestingly, environments that affect protein folding and hence Ura3p activity can directly influence the supply of nucleotides in a cell (Fig 3D). Moreover, the environment can also affect the cellular state and the growth rate and cause changes in the cellular demand for Ura3p activity (e.g., low temperature reduces speed of cell division, hence DNA replication and, thus, rate of nucleotide consumption; Fig 3D, right panel). In this context, it has been demonstrated that the growth rate affects cell fitness and the sensitivity to environmental stresses; for instance, rapidly growing cells tend to be more sensitive to antibiotics than slow‐growing or stationary cells (Berney *et al*, 2006; Lu *et al*, 2009). Thus, specific environments can influence protein folding, and hence the total amount of free/deposited protein, and modulate the cell state by influencing the supply/demand for a particular biochemical activity (Fig EV4). In this way, different environments can modulate the magnitude of the fitness costs and benefits of sequestering Ura3p_agg_ in deposits.

Overall, these results help explain why the phase separation of a single protein can lead to different selection coefficients in the different environments. Specifically, in our model protein, cell fitness primarily relies on the balance between the supply/demand of Ura3p activity and to a much lesser extent on the cost of deposit formation. This means that when uracil is present in the media, the cell does not require Ura3p (no demand). Hence, a change in the amount (free/deposited) or quality of this enzyme (active, folded/inactive, misfolded) in a specific environment does not affect cell fitness significantly. In agreement with this possibility, in these environments, there is no correlation between the fraction of the deposited protein (or free protein) and the selection coefficient (Fig 3B, first and second panels, gray dots). However, when the protein activity is essential or toxic (i.e., in the absence of uracil or presence of 5FOA), each environmental condition has an associated Ura3p demand. Hence, the amount of free protein (ratio of F_CYTOagg_/F_TOTALsol_, in other words the Ura3p supply) as well as the quality of the enzyme determines the overall fitness effect (Fig 3C and D).

Given the extensive variability in fitness of a strain expressing a protein that forms deposits in different environments (Figs 2 and 3), we computationally investigated how the environment could influence the population frequency of variants carrying this protein at the population level.

### History of the environments determines population frequency of strains that can form phase‐separated deposits

**Figure 4:**
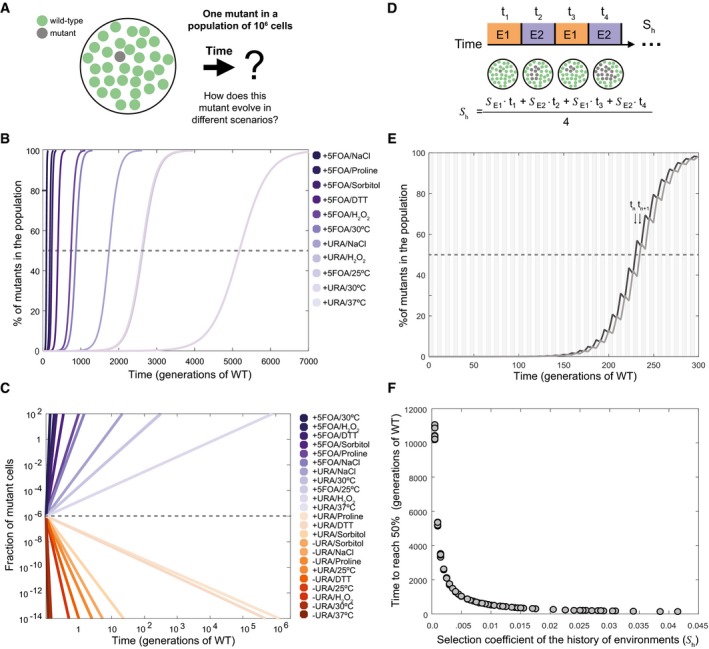
Computational simulation and estimation of population frequencies Schematic of the question addressed for the computational simulation.Population frequency of the phase separation‐prone variant over time (in number of generations with respect to wild‐type strain at 30°C, 2.7 h) in different non‐oscillating conditions with a positive measured selection coefficient (*S*) for protein phase separation. A dashed line indicates the point where the mutant is present in 50% of the population. The corresponding value in the *x*‐axis for each profile denotes the time required to reach this point.Population frequency of the phase separation‐prone variant over time in different environmental conditions with positive and negative *S*. The dashed line indicates the initial fraction of mutant cells in the population, and the colored lines indicate the fraction of mutant cells in the population at different time points (generations of WT is used as the unit of reference).Diagram showing how the frequency of the mutant cells can change in fluctuating environments (environment, E, and time in environment, t). The sequence of environments (history) experienced by a mutant determines the overall selection coefficient (*S*_h_).Population frequency of the variant over time in two different alternating environments. Black line, profile of population frequency of the variant, where, at the moment in which the first mutant cell appears, the environment positively selects it; however, in the subsequent environment the selection is negative. Gray line, profile of population frequency of the variant, where, at the moment in which the first mutant cell appears, the environment negatively selects it; however, in the subsequent environment the selection is positive. The area of the plot is divided into gray (E1) and white (E2) sections to indicate the fluctuating environment with positive and negative *S*.History of environments resulting from any possible combination of two environments (e.g., “environment 1” 25°C and “environment 2″ 0.5 M proline), from those experimentally tested in this work (actual data available in Appendix Table S3).

Our experiments were performed with a 1:1 population (URA3_agg_:URA3_sol_), grown in an exponential phase and with no resource limitation. However, in nature, variants that can form phase‐separated structures arise sporadically in an already growing population, possibly in more severe conditions (Newby & Lindquist, 2013). To investigate how such a variant changes in frequency with time in a population, and whether such a variant can take over the population in a defined timescale, we developed a computational model and simulated a scenario where one individual in a population of a million cells acquires a mutation that results in the formation of protein deposits (Fig 4A; Materials and Methods). Using the experimentally measured selection coefficient values (*S*) (Appendix Table S1), we followed how an individual that carries a protein prone to form deposits is selected for, or against, in a cell population in diverse scenarios. From the simulations, we infer that in a stable environment and when deposit formation is beneficial, the variant can become a dominant member of the population (i.e., more than 50%) within ~50–5,000 generations (Fig 4B and C, blue color range; Appendix Table S2). As expected, if protein phase separation is detrimental to cell fitness in an environment, the variants containing the protein that form deposits never achieve population frequencies higher than the starting condition and get “diluted” with time (Fig 4C, orange color spectrum).

In nature, however, the growth environment can fluctuate more or less rapidly over time (e.g., day/night cycles affect growth temperatures daily; Newby & Lindquist, 2013). This means that an individual with a protein variant that can phase‐separate can be selected to different extents as determined by the sequence/history of environments (*S*_h_) that the population experiences (Kussell & Leibler, 2005; Leibler & Kussell, 2010; Fig 4D and Appendix Table S3). In rapidly fluctuating and alternating environments, as long as the average selection coefficient over time (or selection coefficient of the history of environments, *S*_h_) is positive, the frequency of the phase separation‐prone variant in the population becomes higher over time (Fig 4E). It is worth noting that the frequency of the variant becomes higher irrespective of whether the immediate environment when the mutation is acquired is beneficial (Fig 4E; dark line) or detrimental (Fig 4E; light line) to fitness, as long as the environment fluctuates and *S*_h_ is positive. Nevertheless, at a given time, the frequency of the phase separation‐prone variant can be higher if the mutation is acquired in a favorable environment compared to a detrimental environment (Fig 4E; dark line vs. lighter line at *t*_*n*_ and *t*_*n*+1_). These simulations highlight that for the same population, the history of environments (*S*_h_) can determine the persistence and frequency of a phase separation‐prone variant (Fig 4F).

### Protein phase separation: a source of phenotypic variability

**Figure 5:**
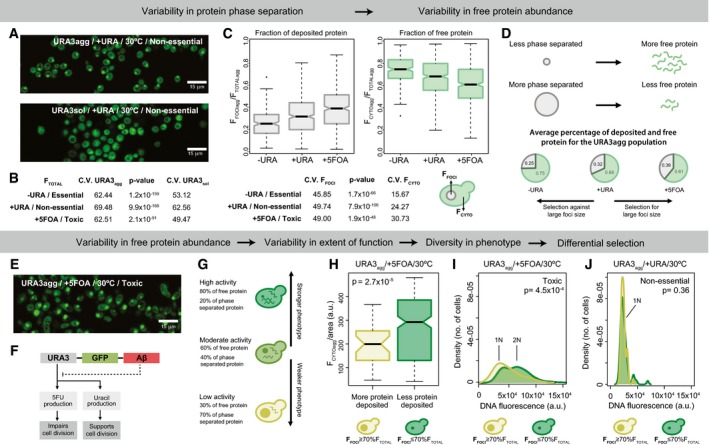
Impact on cell‐to‐cell variability and phenotypic diversity Panels (A–D) show that the variability in foci formation contributes to variability in free protein abundance.
AConfocal microscopy images of URA3_agg_ and URA3_sol_ incubated for 18 h at 30°C.BCoefficient of variation and *P*‐values (Wilcoxon test) of the total fluorescence measured from populations of URA3_agg_ and URA3_sol_.CBoxplot showing the distribution of foci fluorescence (left) or cytoplasmic fluorescence (right), from the foci containing subset of a population of URA3_agg_ grown at 30°C. Coefficient of variation and *P*‐values of each boxplot are shown below.DSchematic showing how the formation of protein deposit can affect the abundance of free protein in a cell. Pie charts showing the average fraction of deposited and free protein of URA3_agg_ in different media compositions and at 30°C.EConfocal microscopy image of URA3_agg_ incubated in the presence of 5FOA after 18 h at 30°C.FSchematic showing that in our system, the phase separation‐promoting region can modulate Ura3p activity and thus its phenotypic effect in a cell.GSchematic highlighting that higher/lower level of free protein inside the cell results in higher/lower Ura3p activity leading to the manifestation of a stronger/weaker phenotype.HBoxplots of the cytoplasmic fluorescence normalized by the cell area (F_CYTOagg_/cell area) for individual cells of the URA3_agg_ population in 5FOA. Light green box, cells with foci containing at least 70% of the total fluorescence (big foci). Dark green box, cells with foci containing less than 70% of the total fluorescence (small foci).I, JCell cycle arrest phenotype. Density plots showing the fluorescence distribution of individual cells from the URA3_agg_ population stained with propidium iodide to measure their DNA content. The cells were incubated for 18 h at 30°C without (I) and with 5FOA (J).Data information: (C, H) For the boxplots: the central line represents the median, the notches represent 95% confidence interval of the median, the box shows the interquartile range (IQR), the whiskers add 1.5 times the IQR to the 75 percentile (box upper limit) and subtract 1.5 times the IQR from the 25 percentile (box lower limit). We used the Wilcoxon test to compare samples.

Microscopy image analysis reveals the existence of stochastic, cell‐to‐cell variation in total protein abundance (i.e., expression noise Soboleski *et al*, 2005; Acar *et al*, 2008; Jothi *et al*, 2009; Burga *et al*, 2011; Chevin, 2011; Ravarani *et al*, 2016) as measured by GFP fluorescence; Fig 5A, Appendix Fig S3). Under standard growth conditions (in which both strains present similar transcript levels; see Fig EV2), we find that the cell‐to‐cell variation in protein abundance is higher for URA3_agg_ compared to URA3_sol_ irrespective of whether Ura3p activity is essential, non‐essential, or toxic (as measured by the coefficient of variation; CV = 100·σ/μ; ratio of the standard deviation to the mean abundance; Fig 5B). However, if we focus on just one genotype, regardless of URA3_sol_ or URA3_agg_, we obtain higher noise when uracil is added. This is consistent with the current view that the maximum population variance is achieved under conditions with no selection (Thompson & Cubillos, 2017). Interestingly, the cell‐to‐cell variation in protein abundance is higher in the deposits compared to the cytosol for URA3_agg_ irrespective of whether Ura3p is essential non‐essential or toxic (Fig 5C). These data suggest that the extent of protein deposit formation by phase separation can modulate the abundance of free/active protein in a cell (Fig 5D). We find that when Ura3p activity is essential or toxic, on average, cells with a higher fraction of phase‐separated protein are selected against (i.e., higher free protein is selected for) or for (i.e., less free protein is selected for), respectively (Fig 5D). When Ura3p is not essential, the average foci size is in between what is observed for the other two growth media, suggesting no obvious selection for foci size (or free protein) (Fig 5C). These results indicate that even within an isogenic population, the extent of deposit formation between individuals can be selected for or against under different selective pressures. This effect is similar to what one observes in prion strain selection when yeast cells are grown under different stress conditions (Holmes *et al*, 2013; Chakrabortee *et al*, 2016).

In conditions where Ura3p activity is toxic, we observe that cells are elongated, tend to be larger, and display cell division problems (Fig 5E). This is consistent with the fact that in the presence of 5FOA, free/active Ura3p produces 5FU and leads to cell cycle arrest during DNA synthesis (Fig 5F; Seiple *et al*, 2006). Since the formation of phase‐separated structures reduces the amount of free/active protein (Fig 5D), individuals with larger amount of protein in deposits (F_FOCI_ > 70% F_TOTAL_) will have little free protein and display less Ura3p activity (Fig 5G), and hence should less often exhibit cell cycle arrest. Similarly, individuals with smaller amount of protein in deposits will display higher Ura3p activity (Fig 5G), and hence should more often exhibit cell cycle arrest. In line with this expectation, we observe that within the same population, individual cells with more protein recruited into phase‐separated deposits tend to have less free protein after normalizing for cell area (Fig 5H). Furthermore, DNA quantification reveals that cells with more protein in phase‐separated deposits tend to display cell cycle arrest less often compared to those with less protein in deposits (Fig 5I). Indeed, in conditions where Ura3p activity is not essential, no cell cycle arrest is observed (Fig 5J).

**Figure 6:**
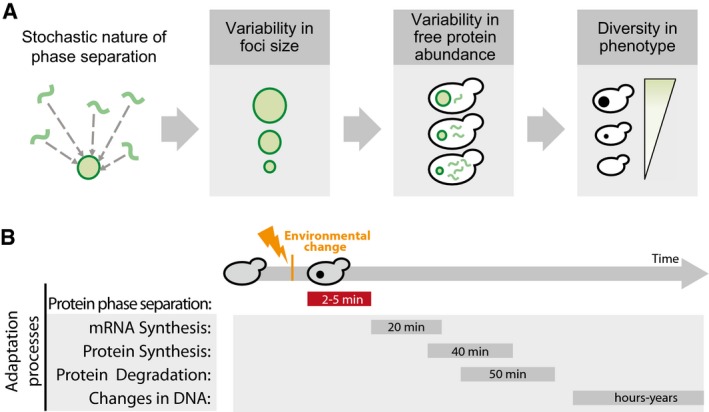
Phenotype modulators The stochasticity of deposit formation due to phase separation will define the foci size, which in turn will influence the amount of free protein in the cytosol. The abundance of the free protein (or the phase‐separated structure) will ultimately determine the cellular phenotype. The behavior and phenotypic outcome may also be influenced by the molecular structure and properties of the phase‐separated structure (e.g., complex coacervates, liquid droplets).Timeline for the different processes that are important for adaptation/response to an environmental change. Time estimates were obtained from published datasets (e.g., Wang *et al*, 2002; Belle *et al*, 2006; Escusa‐Toret *et al*, 2013).

**Figure EV5:**
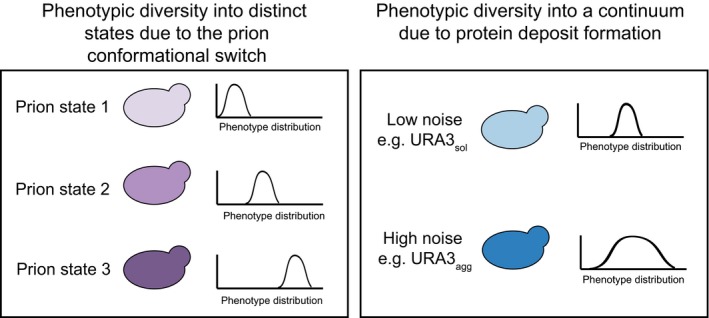
Population diversity introduced by prion conformational switching and by deposit formation Prion conformational switching is associated with the emergence of different prion strains that provide new heritable phenotypes with distinct and largely non‐overlapping phenotypes or functional states, whereas, a more general protein deposit formation upon phase separation may create a phenotypic continuum.

Taken together, our results suggest that the cell‐to‐cell variability in the extent of protein phase separation can lead to variability in the abundance of free, functional protein, which in turn can generate a phenotypic continuum in a genetically identical cell population (Fig 6A). It should be noted that depending on the molecular structure and the properties of the phase‐separated assembly (e.g., insoluble deposits or liquid droplets), one could find an opposite behavior where enzyme activity may be higher within certain types of phase‐separated structures (Shin & Brangwynne, 2017). Nevertheless, such phenotypic diversity, induced by variable protein phase separation, can provide a selective advantage under certain conditions and could enhance the likelihood of survival of some individuals from a population after an environment alteration. Similar benefits have been attributed to the prion conformational switch. An important difference, however, is that in the latter case, instead of generating a phenotypic continuum, the emergence of different prion strains provides new heritable phenotypes with distinct and largely non‐overlapping phenotypes/functional states (Derdowski *et al*, 2010; Halfmann *et al*, 2010; Holmes *et al*, 2013; Newby & Lindquist, 2013; Farkash‐Amar *et al*, 2014; Chakrabortee *et al*, 2016; Newby *et al*, 2017; Fig EV5). Our observations suggest that beyond prions, polypeptide chains with certain phase separation/aggregation/deposit‐forming propensity can contribute to selectable phenotypic heterogeneity to an isogenic cell population. In other words, the selectable phenotypic variability described for prions is likely to be a general property of any polypeptide segment that can form phase‐separated protein deposits.

## Discussion

Our understanding of the process of protein phase separation has changed rapidly in the last decade. Currently, there is consensus that intracellular protein condensates are associated with a wide range of effects, ranging from pathological to beneficial (Aguzzi & Altmeyer, 2016). Clearly, not all deposits have the same molecular structure (Brangwynne *et al*, 2015; Molliex *et al*, 2015; Zhang *et al*, 2015; Feric *et al*, 2016; Lee *et al*, 2016). In the cellular milieu, the different types of deposits differ in their composition, dynamics, and cellular location. This in combination with the biochemical function of the molecules that participate in phase separation determines the overall effect on fitness (Morley *et al*, 2002; Gsponer & Babu, 2012; Lin *et al*, 2015; Miller *et al*, 2015; Molliex *et al*, 2015; Zhang *et al*, 2015; Zhu & Brangwynne, 2015; Banani *et al*, 2016; Feric *et al*, 2016; Mitrea & Kriwacki, 2016; Pak *et al*, 2016; Chavali *et al*, 2017b; Riback *et al*, 2017). To investigate how phase‐separated protein deposits can affect fitness and to disentangle the effects of different factors on cell fitness, we studied a model protein with a modular architecture (Fig 1B) that is prevalent in nature (Bornberg‐Bauer & Alba, 2013; Lees *et al*, 2016; Chavali *et al*, 2017a). Such a protein architecture may be widespread, possibly because it allows the properties of the polypeptide segment that promotes phase separation (e.g., concentration at which the protein forms deposits) to evolve independently of the biochemical function that is mediated by the rest of the protein. Proteins without this modular architecture may also tune their aggregation propensity; however, since function and aggregation propensity are linked in such proteins, evolution of their sequences will likely be more constrained.

Our work is consistent with previous studies (Geiler‐Samerotte *et al*, 2011; Escusa‐Toret *et al*, 2013; Tomala *et al*, 2014) and provides quantitative insights as well as a framework to decouple the fitness cost and benefit of protein phase separation, and the associated loss/gain of protein function in different environments. Overall, the data suggest that in our system, yeast cells can tolerate and are adapted to intracellular protein phase separation of the model protein that we have designed. In future, this modular architecture can be adapted to assess the specific contribution of different types of phase separation‐promoting sequences on cell fitness. In our system, the effect on fitness is primarily driven by either the loss or gain of protein function as a consequence of sequestering the free/active/functional protein into insoluble deposits (Fig 3C). Furthermore, the environment can modulate the consequences of protein phase separation in strains with the same genomic background, enabling situations where protein deposit formation is either beneficial or detrimental to fitness (Fig 2C). These results indicate that the interplay between the protein state (e.g., soluble or deposited) and the functional demand placed by the cell defines the overall effect of protein phase separation on cell fitness. It also highlights that the specific growth environment is a key determinant of the effect of phase separation on cell fitness. Using computational simulations, we further showed that the frequency and persistence of a phase separation‐prone variant within a population are determined by the history of environments experienced by this population (Fig 4G). In fact, it has been observed in nature that the history of environments (memory) can be “encoded” by aggregation‐prone Q/N‐rich segments (Wiltzius *et al*, 2009; Caudron & Barral, 2013; O'Connell *et al*, 2014; Zhang *et al*, 2015).

Our observations on individual cells in an isogenic population indicate that the extent and onset of protein phase separation may vary between cells (Fig 5). While this may be influenced by different cellular processes (Chalancon *et al*, 2012), protein phase separation might further contribute to changes in free protein level and leads to cell‐to‐cell heterogeneity in free protein abundance. The existence of cytoplasmic chaperones that can dissolve protein macromolecular assemblies such as Hsp104p (Duennwald *et al*, 2012), and variability in their abundances between cells, could further contribute to the ability of the phase separation‐prone protein to be released or refold from deposits to become active. Such mechanisms that generate cell‐to‐cell variability in abundance can act in synergy and still be distinct from the ability of chaperones to help fold (i.e., buffer) deleterious mutants during or after protein synthesis (Burga *et al*, 2011), leading to stochastic changes in protein abundance between individuals.

A population should generate enough phenotypic variability to ensure that some cells will be able to survive an unexpected environmental change (Kussell & Leibler, 2005; Koonin, 2007; Acar *et al*, 2008; Leibler & Kussell, 2010). In this sense, like in the case of a prion conformational switch, phase separation can be a general mechanism of adaptation by resulting in rapid and transient manifestation of loss/gain of function compared to mechanisms that involve changes in the mRNA, or protein levels (Wang *et al*, 2002; Kussell & Leibler, 2005; Belle *et al*, 2006; Leibler & Kussell, 2010; Escusa‐Toret *et al*, 2013; Fig 6B). In this context, variation in the formation of deposits, and variable stoichiometry of the deposits between individuals of a population can further generate phenotypic diversity (Fig 6A). In other words, since deposit formation is dependent on protein concentration (Veis, 2011; Ciryam *et al*, 2013; Brangwynne *et al*, 2015), different individuals can form phase‐separated assemblies at different abundances by “sensing” their concentration in cells (Gsponer & Babu, 2012). This may lead to variable and rapid manifestation of phenotypes by transiently sequestering proteins into deposits (or restricting their localization in a cell) in different environments. Interestingly, in most cases this phenomenon is triggered because proteins have cellular concentrations above their saturation level (Ciryam *et al*, 2013). For these supersaturated proteins, formation of such deposits can be protective when toxic concentrations are reached in a cell (Bershtein *et al*, 2012).

The modular nature of the phase transition‐promoting segment also helps with phenotypic diversity and the manifestation of new phenotypes during evolution, since such segments can evolve independently of the biochemical function mediated by the rest of the polypeptide (Bornberg‐Bauer & Alba, 2013; Boke *et al*, 2016; Lees *et al*, 2016). This may possibly explain why many regulators such as transcription factors (e.g., Snf5) contain phase separation‐promoting segments (e.g., Q/N‐rich segments) independently of structured domains that perform specific biochemical function such as DNA binding (Chavali *et al*, 2017a; Chong *et al*, 2018; Sabari *et al*, 2018). It is known that cell‐to‐cell variation in protein abundance of such regulators can lead to differential activation of downstream regulatory networks and drive the phenotypic differences in an isogenic population (Jothi *et al*, 2009; Halfmann *et al*, 2012; Holmes *et al*, 2013; Gemayel *et al*, 2015). Thus, variability in deposit formation between individuals can lead to cell‐to‐cell differences in phenotypes by affecting the abundance of the free/active protein, as observed with foci size variability, and cell cycle arrest phenotype in the URA3_agg_ population (Fig 5).

The findings presented here suggest that in addition to variability in the process of gene expression, and protein folding between individuals of a population (Kussell & Leibler, 2005; Acar *et al*, 2008; Jothi *et al*, 2009; Leibler & Kussell, 2010; Burga *et al*, 2011; Chevin, 2011; Holmes *et al*, 2013; Ravarani *et al*, 2016), phase separation‐induced cell‐to‐cell variability in free protein abundance is likely to be an important phenomenon that can facilitate adaptability in different conditions. Thus, studies on the effects of phase‐separated structures such as intracellular protein deposition should not only involve a global investigation of the cost and benefit but also consider cell‐to‐cell variability of phenotypes in the population (Farkash‐Amar *et al*, 2014).

Inside the cell, a myriad of proteins phase‐separate by different mechanisms. However, the cellular complexity does not end here since these assemblies can interact with each other, and with other molecules and are subjected to a constant transformation due to the quality control machinery (Farkash‐Amar *et al*, 2014; Brangwynne *et al*, 2015; Lin *et al*, 2015; Nott *et al*, 2015; Zhang *et al*, 2015; Zhu & Brangwynne, 2015; Banani *et al*, 2016; Bolognesi *et al*, 2016; Feric *et al*, 2016; Pak *et al*, 2016; Wu & Fuxreiter, 2016). Overall, in an *in vivo* system, phase separation can affect the cellular processes at multiple levels and result in different fitness costs and benefits. In our system, the protein model phase‐separates from a largely soluble active state to a primarily insoluble inactive state. Obviously, in more complex systems (e.g., dynamic assemblies, active compartments, complex coacervates) there are more variables to be studied than those analyzed here. However, the quantitative insights disentangling the various effects, as well as the conceptual and methodological framework presented in this study, may be adapted (e.g., by testing phase separation‐promoting sequences with different molecular structures and properties, adding new elements, and in different genetic backgrounds) to understand the effect on cell fitness of phase separation of diverse proteins and how this determines their selection within a population.

## Materials and Methods

### Strains and vectors

All the strains employed in this work are based on Y03157 (BY4741; Mat a; his3D1; leu2D0; met15D0; ura3D0; YBR020w::kanMX4) obtained from Euroscarf deletion collection. In this strain, the GAL1 gene is missing, thereby hindering cells to consume galactose (the inducer) as a carbon source (Geiler‐Samerotte *et al*, 2011).

A multicloning site flanked by a 65‐bp region that is homologous to TRP1 (TRP start vector) was designed and introduced in a pMA vector (GeneArt^®^ Life Technologies) (Appendix, Vectors and Primers). The TRP1 locus was selected as the insertion position for the proteins (Ura3p_sol_ and Ura3p_agg_), because of its close proximity to the centromere and the high expression levels of genes in this region. These properties minimize the frequency of gene silencing and ensure a steady state expression level through several generations. A yeast‐optimized GFP codon and the SpHis5 selection marker were extracted from a pKT128 vector (Sheff & Thorn, 2004) and inserted between the restriction sites PacI and SacI. The pGAL1 promoter was obtained from a pESC‐URA vector (Agilent Technologies) and inserted between HindIII and SalI. The same vector was used to extract the locus encoding the URA3 gene plus an additional 12‐residue linker (Linker 1: GGTACCGCTAGTGGTTCTGCTGGTTCTGCGATTAAC). URA3 was inserted between pGAL1 and GFP using the In‐Fusion^®^ HD Cloning Kit (Clontech) (TRP‐URA vector).

The sequence of Aβ42 was optimized for yeast using the codon usage reported in the Codon Usage Database (http://www.kazusa.or.jp/codon/) and was built using four DNA oligos. Aβ42 was inserted 3′prime of the GFP (TRP‐URA‐AB vector) using the In‐Fusion^®^ HD Cloning Kit (Clontech) and included a 12‐residue linker in between (Linker 2: GGTGGAAGTGCTAATGGTACTTCTGGTGCTAGTGGT).

Both linkers were designed to be yeast codon optimized and enriched in small amino acids to provide flexibility and avoid steric hindrances. Additionally, these sequences have a low net charge and no aggregation‐prone regions to prevent interference with the phase separation process triggered by Aβ42. This second property was measured using the AGGRESCAN algorithm (Conchillo‐Sole *et al*, 2007; de Groot *et al*, 2012). The cellular behavior of Aβ42 has been extensively studied in bacteria and yeast, both by others and by us (de Groot & Ventura, 2006; de Groot *et al*, 2006; Morell *et al*, 2011; Villar‐Pique *et al*, 2012; Sanchez de Groot *et al*, 2015). In yeast, the formation of deposits/granules appears to protect cells against the toxic species (e.g., soluble oligomers; de Groot & Ventura, 2006; Villar‐Pique & Ventura, 2013; Sanchez de Groot *et al*, 2015).

### Rationale and design of model proteins

In this work, we designed model proteins to study the effects of protein phase separation on cell fitness. With this aim, we designed a polypeptide with a modular arrangement, similar to those recently found in nature (McGlinchey *et al*, 2011; Kato *et al*, 2012; Kedersha *et al*, 2013; Sleeman & Trinkle‐Mulcahy, 2014; Fig 1C). This setup allows us to tune different properties while studying the cost/benefit of the phase separation event (see main text).

To build our model proteins, we chose the yeast endogenous Ura3p enzyme as the functional domain. This provides a versatile framework for assessing the different roles of the enzyme, as the biochemical activity can be essential, non‐essential, or toxic depending on the composition of the growth medium.

To report the expression, distribution, and location of the fusion protein, we added the enhanced GFP (F64L/S65T) as the reporter. This GFP variant includes a mutation at the position 64 (F−>L) that allows its correct production at 37°C (Cormack *et al*, 1996; Day & Davidson, 2009). This extra stability is crucial for the present work to ensure the integrity of the reporter under stress conditions. Importantly, as reported in earlier studies in *E. coli*, even under strong overexpression, the folding of this enhanced GFP fused with phase separation‐promoting peptide is faster than the phase separation process itself. This property results in the formation of fluorescent foci at a temperature range between 25 and 37°C (de Groot & Ventura, 2006; de Groot *et al*, 2006). Furthermore, to distinguish between the effect of the environment and foci formation on the steady state abundance of the model protein (GFP fluorescence), our system includes a soluble control (URA3_sol_) to indicate the maximum amount of active GFP that can be made available in a cell at a certain environment. Hence, in the unlikely event in which a GFP molecule will misfold due to reasons not associated with the phase separation process, the normalization against the control strain will account for such a situation.

Finally, Aβ42 was added as a tag to promote phase separation and study the effect of deposit formation. This peptide is the main component of the plaques found in patients with Alzheimer's disease (Chiti & Dobson, 2006; Olzscha *et al*, 2011; Ciryam *et al*, 2013; Pak *et al*, 2016; Woerner *et al*, 2016). *In vitro*, Aβ42 assembles into amyloid fibrils driven by its hydrophobic residues. When overexpressed in unicellular models, this peptide accumulates into inclusion bodies (de Groot & Ventura, 2006; de Groot *et al*, 2006; Plata *et al*, 2010; Morell *et al*, 2011; Villar‐Pique *et al*, 2012; Escusa‐Toret *et al*, 2013; Villar‐Pique & Ventura, 2013; Sanchez de Groot *et al*, 2015; Pak *et al*, 2016). Aβ42 has no intrinsic function in *S. cerevisiae* and, according to our previous studies, its expression has almost no effect on yeast growth, at least under optimal grow conditions (Villar‐Pique & Ventura, 2013; Sanchez de Groot *et al*, 2015). Consistent with this, it is worth emphasizing that we measured no significant difference in growth that was associated with the addition of Aβ42 in conditions where Ura3p is non‐essential and no stress is introduced (Fig 2). An alternative strategy to induce the phase separation of Ura3p could be the alteration of its sequence with the aim to destabilize the protein and to induce foci formation. However, this mutant variant can also affect Ura3p structure and enzymatic activity, increasing the complexity of the system and making it more difficult to determine the origin of the cell fitness alterations. We also considered the addition of a less aggregation‐prone Aβ42 peptide; however, all point mutations studied have a similar growth rate (Villar‐Pique & Ventura, 2013; Sanchez de Groot *et al*, 2015), and to obtain a fully soluble variant, multiple mutations are necessary (Wurth *et al*, 2002). Therefore, to generate the soluble version of the model protein, we used the version without a tag. In short, we designed the model protein in a modular manner with the addition of a phase separation‐promoting tag (i.e., separate functional and aggregation‐prone region in the same polypeptide).

With the above‐mentioned elements, we built two different constructs: (i) One was designed to control for the intrinsic fitness cost of overexpressing a soluble protein (Plata *et al*, 2010; Geiler‐Samerotte *et al*, 2011; Kafri *et al*, 2016) (Ura3p fused to GFP, Ura3p_sol_), and (ii) the other one was designed to investigate the effect of protein deposit formation (Ura3p fused to GFP and Aβ42 peptide, Ura3p_agg_, Fig 1D). By comparing the overall cell fitness upon expressing these two constructs, we obtain the fitness effects of cells expressing the phase‐separated Ura3p_agg_. It is worth mentioning that the obtained phenotype is not that of a full null strain. The microscopy images demonstrate that there is still cytosolic fluorescence in the Ura3p_agg_ strain (Figs 5 and EV1). These data suggest that the fitness effects associated with deposit formation of Ura3p are smaller than those obtained with a true auxotroph (Mulleder *et al*, 2012).

For the mathematical modeling of this system (see Selection coefficient calculation), we have considered some generalizations for the analysis of the different parameters influencing the phase separation event and its effect on cell fitness. We have subtracted the protein exchange between the phase‐separated assembly and the surrounding milieu from our analyses based on two experimental observations. First, FRAP analyses show that Ura3p_agg_ forms a stable, non‐dynamic deposit similar to IPODs (Fig EV3). Second, our analyses are done keeping the culture at exponential phase and after long expression times (from 18 h to 3 days). This setup allows Aβ42‐GFP deposits to reach an equilibrium in which most cells contain just one big focus (see Fig 5) and the amount of protein synthesized/degraded appears to be constant (Morell *et al*, 2011). Thus, in these conditions we consider that the cells reach a “population equilibrium” in which some properties of the culture are constant: (i) The number of newborn cells is constant (exponential phase); (ii) the cells are adapted to the environment (after 18–72 h), and therefore, the environmental pressure/selection is constant; and (iii) considering assumptions (i) and (ii), the noise distribution of Ura3p expression and the number of foci‐forming cells in the population remain stable (Morell *et al*, 2011; Sanchez de Groot *et al*, 2015). This premise also considers that the cell‐to‐cell heterogeneity in foci formation is a final outcome of the inherent variability of cellular processes such as protein expression and quality control activity (e.g., chaperones; Chalancon *et al*, 2012; Farkash‐Amar *et al*, 2014; Ravarani *et al*, 2016).

Under the microscope, Ura3p_sol_ seems homogeneously distributed throughout the cytosol whereas part of Ura3p_agg_ is accumulated into foci. It has been reported that not all the protein in the soluble fraction is active (e.g., soluble oligomers) and that not all the aggregate protein is inactive (i.e., fluorescence into foci; Villar‐Pique & Ventura, 2013). However, at least in our system, due to the static nature of the analyzed foci, the Ura3p recruited in them will have limited access to its substrate (irrespective of its conformation and functional state). In addition, theoretically, the free Ura3p should have less constraints to acquire a correct fold than the protein located in the foci (Morell *et al*, 2011). Overall, based on these premises, we assume that the protein located in the cytosolic fraction is more active and accessible to substrate than the protein located in the assembly, so we consider that the residual activity located in the foci is negligible. It should be noted that depending on the nature of the phase‐separated structure (e.g., liquid droplets), one could find an opposite behavior where enzyme activity may be higher within certain types of phase‐separated structures (Shin & Brangwynne, 2017).

### Media composition and environmental conditions

In all the assays, the strains were grown in SD ‐HIS media containing a mixture of sugars and amino acids. Since protein concentration is a critical factor for foci formation, to guarantee high levels of expression we measured the effect of galactose concentration (the inducer) on URA3_sol_ expression by monitoring the GFP fluorescence using flow cytometry (Appendix Fig S2D). Maximum levels of expression were obtained between 0.5 and 2%. Below 0.5% of galactose, the fluorescence declines abruptly, so we chose 1% as a reliable concentration to keep high levels of induction. To obtain a fresh culture before inducing Ura3p expression, single colonies were picked and grown overnight in 2% glucose. This culture was employed to inoculate SD ‐HIS in 2% raffinose and grown for 6 h. Then, it was inoculated in fresh media with 2% raffinose and 1% galactose. SD ‐His ‐Ura was employed to test the essentiality of Ura3p. SD ‐His with uracil and 5FOA (Zymo Research) was used to analyze the phase separation effects of a toxic Ura3p activity.

In all cases, the media were adjusted to pH 4.5 to ensure (when required) the 5FOA permeability. To test different environments, the different compounds were added before the pH adjustment. The final concentrations tested are 0.5 mM H_2_O_2_, 1.5 mM DTT, 0.5 M NaCl, 1 M sorbitol and 0.5 M proline. The cells were grown at 220 rpm and 30°C before starting the experiments to test the effect of a specific environment. Subsequently, the cultures were incubated at 30° with the corresponding concentration of the above‐mentioned chemical or without the chemical but shifting the temperature to 25° or 37°C.

### Cell cycle arrest

Cells grown until exponential phase were fixed by incubating them for 1 h with ethanol 70% at 4°C. Then, the cells were treated with 1 mg/ml RNase A (Sigma) for 2 h at 37°C. The samples were stained with propidium iodide (50 μg/ml) for 1 h before analyzing them under a confocal microscope. The fluorescence of propidium iodide was employed as a measure of DNA content and an indication of the cell cycle arrest.

### Confocal microscopy

Cells were grown at exponential phase with 1% galactose and the corresponding environmental condition for 18–20 h before acquiring the images. The images were acquired with a Zeiss 710 (Carl Zeiss) with an objective of 63×, an excitation laser of 488 nm, and emission window between 581 and 750 nm. The cells expressing soluble Ura3p (Ura3p_sol_) displayed fluorescence that is homogeneously distributed through the cytoplasm, whereas the cells that additionally express the Aβ (Ura3p_agg_) showed fluorescence in foci. At least 100 cells were captured for each strain and environment. For the propidium iodide assay, the fluorescence was excited with 561 nm and the emission was collected between 566 and 719 nm.

### Image processing

All the images were processed with Fiji (ImageJ). For each cell, the program measured the cell area, the cytosolic fluorescence, and the fluorescence contained in the aggregates. A macro was created to perform these measures automatically. The parameters of this macro for a whole cell analyze the presence of elements with (i) a fluorescent intensity between 20 and 255 a.u., (ii) an area (size) between 8 and 50 μm^2^, and (iii) a circularity between 0.25 and 1. To detect foci, a mask was generated including the elements with a fluorescent intensity between 40 and 255 a.u. and an area between 0.2 and 10 μm^2^. For the cells expressing Ura3p_agg_, the fluorescence from the foci was subtracted from the total fluorescence to obtain the amount of soluble protein in each cell. The cell size analysis shows a positive correlation with the foci size (Appendix Fig S3), probably due to aging and yeast asymmetric division, which retains protein deposits in mother cells (which tend to bigger) producing clean daughter cells (which tend to be smaller) where there protein assembly has to start *de novo* (Coelho *et al*, 2014). For the propidium iodide assay, we adapted the macro to additionally measure the fluorescence in the propidium iodide channel for each element detected.

### Immunochemistry

Yeast cells were grown for 18 h in media with 2% raffinose and 1% galactose. 20 ml of culture was divided in two and centrifuged. For total fraction, a pellet was resuspended in 75 ml of lysis buffer (10 mM Tris–HCl, pH 1/4 8, 150 mM NaCl, 0.05% Tween‐20, 10% glycerol, 5 mM EDTA, 1 mM DTT, 2 mM PMSF) and 25 ml NuPAGE LDS sample buffer with 2.5% 2‐mercaptoethanol (w/w) and incubated at 100°C for 5 min. For fraction separation, a pellet was resuspended in 75 ml Y‐PER yeast protein extraction reagent (Thermo Scientific) supplemented with 0.1 mM PMSF and incubated at room temperature with agitation for 20 min. Then, the sample was centrifuged to separate the two fractions: soluble (supernatant) and insoluble (pellet resuspended in 75 ml of PBS). 25 ml of NuPAGE LDS sample buffer with 2.5% 2‐mercaptoethanol (w/w) was added to both fractions and then incubated at 100°C. To separate the proteins, 5 ml of the total fraction and 10 ml of soluble or insoluble fractions were eluted into a precast NOVEX NuPAGE 4–12% gels in denaturing conditions. The Invitrogen iBlot system was used to transfer proteins to PVDF membranes. After blocking, membranes were incubated overnight at 4°C with anti‐GFP rabbit antibody (Santa Cruz sc‐8334) or anti‐PGK1 mouse antibody (Novex 459250) diluted 1:1,000 and 1:10,000, respectively. Secondary incubation with anti‐Protein G HRP conjugate (Millipore 18‐161) at 1:10,000 was performed at RT for 1 h. Images were taken with an Amersham Imager 600. ImageJ 2.0.0‐rc‐48 software was used to quantify protein bands.

### *In vivo* half‐life measurement

Yeast cells were grown for 18 h in media with 2% raffinose and 1% galactose. Protein production was then stopped by cleaning the cells and changing the media to SD ‐HIS with 2% glucose. All samples were adjusted to the same concentration before monitoring the turbidity (absorbance, 600 nm) and fluorescence (excitation 480 nm, emission 510 nm) with a TECAN Infinite 200. Samples were agitated for 10 s before each measurement and during 450 s between time points. The fluorescence was measured as the ratio between fluorescence and turbidity at every time point. To calculate the ratio of fluorescence loss, the data were fitted to a Boltzmann's sigmoid with GraphPad PRISM 5 software (GraphPad Software).

### Competition experiments

Cells pre‐induced with 1% galactose were grown in SD ‐His overnight and then inoculated into fresh media for 4 h to reach exponential phase. Then, the OD_600 nm_ of the cultures was measured, and the two strains were mixed in equal concentration (1:1) and this mixture was employed to inoculate the different media. At this point, we took a sample for time 0. At least two different duplicates of each culture were analyzed. The cells were kept at exponential phase to guarantee a stable duplication time and to minimize the number of old and dead cells. For each culture, samples were collected at six different time points (0, 17, 34, 51, 58, 75, and 82 h). At each time point, the cells were centrifuged and the genomic DNA was extracted. After taking the samples, a dilution was performed to maintain the cultures at OD_600 nm_ between 0.005 and 0.6.

To quantify the proportion of each strain, a pair of oligos (solF/solR and aggF/aggR) was designed to obtain a specific product close to 100 bp (Appendix, Vectors and Primers). The oligos to quantify URA3_sol_ anneal between URA3 and GFP. For URA3_agg_, the primers anneal specifically to the Aβ sequence. The specificity and efficiency of each pair of oligos were tested against the plasmids containing the inserted cassettes (Fig EV2). A control PCR with an empty plasmid (TRP start) produced no amplification product. For each qPCR assay, two standard slopes of plasmids encoding for Ura3p_sol_ and Ura3p_agg_ (pESC‐URA and TRP‐URA‐AB vectors) were included in the same plate to correct for PCR efficiency and measure the number of gene copies (Fig EV2). The ΔΔ*C*_T_ method was employed to analyze each qPCR (Teste *et al*, 2009). Log_2_ of the ratio of the strains (URA3_agg_/URA3_sol_) was plotted. The resultant slope corresponds to the difference between the growth rates of the two strains.

Before the optimization of the above‐described method of growth measurement, we also tried to implement other approaches previously reported as highly efficient to measure the effects of protein misfolding on cell fitness (Geiler‐Samerotte *et al*, 2011). However, several experimental problems appeared after altering the environmental conditions. For example, we were not able to follow by flow cytometry the competition between yeast strains expressing a soluble and insoluble protein variant tagged with different fluorescent markers (i.e., YFP and GFP) at 37°C or 0.5 mM H_2_O_2_. The extreme growth conditions employed influenced the marker's folding and fluorescence spectrum, impeding the correct identification of the populations.

### Doubling time

URA3_sol_ and URA3_agg_ were grown separately in 96‐well plates. The turbidity (OD_600 nm_) and fluorescence (450 nm excitation and 510 nm emission) were recorded at 72 h with a Tecan Infinite M200 Pro. Before starting the assay, the cells were pre‐induced with SD ‐His 2% raffinose–1% galactose overnight and then incubated in fresh media for 4 h to reach exponential phase. The time‐course assay was started with an OD_600 nm_ of 0.02 (Cary 50 Bio). A spline interpolation approach was applied with the RStudio program to measure the slope of each growth curve, resulting in the doubling time τ (Appendix Table S2).

### Selection coefficient calculation

The selection coefficient is defined as in Chevin (Chevin, 2011):

(1) $$Selection=S=\frac{d}{\mathrm{dt}}\ln\left(\frac{P}{1-P}\right)=\frac{d}{\mathrm{dt}}\ln\frac{N_{\mathrm{agg}}}{N_{\mathrm{sol}}}$$

where *P* is the proportion of a certain strain in the population, *N*_*agg*_ is the number of cells of URA3_agg_, and *N*_*sol*_ is the number of cells of URA3_sol_ at time *t*.

The proportion of each strain at a certain time point is:

(2) $$\frac{N_{\mathrm{agg}}}{N_{\mathrm{sol}}}=\frac{N_{\mathrm{agg}}^{0}2^{t\varpi_{\mathrm{agg}}}}{N_{\mathrm{sol}}^{0}2^{t\varpi_{\mathrm{sol}}}}=\frac{N_{\mathrm{agg}}^{0}}{N_{\mathrm{sol}}^{0}}2^{t(\varpi_{\mathrm{agg}}-\varpi_{\mathrm{sol}})}$$

where *N* ^0^ is the initial concentration (concentration at time 0) and ω is the growth rate. The measurement could be simplified using the base 2 logarithm and adding the difference between the growth ratio of the two strains, which can be obtained from the competition experiments:

(3) $$\begin{array}{cc} & \log_{2}\frac{N_{\mathrm{agg}}}{N_{\mathrm{sol}}}=\log_{2}\frac{N_{\mathrm{agg}}^{0}}{N_{\mathrm{sol}}^{0}}+t\left(\varpi_{\mathrm{agg}}-\varpi_{\mathrm{sol}}\right)\;\to \\ & \frac{d}{\mathrm{dt}}\left(\log_{2}\frac{N_{\mathrm{agg}}}{N_{\mathrm{sol}}}\right)=\varpi_{\mathrm{agg}}-\varpi_{\mathrm{sol}} \end{array}$$

(4) $$S=\frac{d}{\mathrm{dt}}\left[\frac{1}{\log_{2}e}\log_{2}\frac{N_{\mathrm{agg}}}{N_{\mathrm{sol}}}\right]\;\;\to \;S=\frac{1}{\log_{2}e}\frac{d}{\mathrm{dt}}\left[\log_{2}\frac{N_{\mathrm{agg}}}{N_{\mathrm{sol}}}\right]$$

By combining (3) and (4), the selection coefficient could be calculated as:

(5) $$S=\frac{1}{\log_{2}e}\left(\varpi_{\mathrm{agg}}-\varpi_{\mathrm{sol}}\right)$$

### Population frequency simulation

The doubling times (τ) employed in the simulations were interpolated from the selection coefficient values (*S*) and the experimentally measured doubling times of URA3_sol_ (the wild‐type) (Appendix Table S1; Appendix Table S2).

To simulate the time evolution of the mutant fraction in a mixed population, we started with a mixed population consisting of one mutant cell (URA3_agg_) and 10^6^ wild‐type cells (URA3_sol_). In the simulation, we used the logarithmic scale to handle large numbers, but the final numbers were converted to linear scale whenever needed. We used an Euler integration scheme (with Δ*t* = 60 min) to numerically calculate number of cells, N (*t*), at any given time, *t*:

(6) $$\;\text{ln}\;\left(N\left(t\right)\right)=\text{ln}\;\left(N\left(t-\Delta t\right)\right)+\frac{\Delta t}{\tau }\text{ln}\;2$$

where τ is the doubling time (τ = 1/ϖ). To simulate alternating environments, we used the same integration scheme as above, but the doubling times were re‐assigned to match a specific environment, i.e., every 12 h. All simulations were performed in MATLAB.

### Measurement of the effect of protein phase separation

In our experiments, the effects of Ura3p phase separation on cell fitness are determined by three factors: (i) the cost of deposit formation, (ii) the cost of losing the essential Ura3p biochemical activity, and (iii) the benefit of gaining a protective function by sequestering Ura3p in the deposit. The effect of foci formation is comprised of the cost due to the sequestration of material and consumption of energy (e.g., amino acid sequestration, ATP‐dependent chaperone activity). The other two factors are associated with protein function that existed before foci formation (loss of function) and the generation of new beneficial effect after foci formation (gain of protection against the toxic function). In our experiments, the loss of function leads to a decrease in pyrimidines (in the absence of uracil), whereas the gain of protective function is due to the prevention of toxic 5‐fluorouracil production (in the presence of 5FOA). We consider that each effect is determined by the amount of phase‐separated protein (F_FOCIagg_/F_TOTALagg_), the amount of soluble protein (F_CYTOagg_/F_TOTALsol_), and a coefficient of proportionality that captures the effect of the specific environment (α, β, γ) (Fig 3, see main text). These coefficients indicate the magnitude of the effect on fitness of forming the deposit and loss and gain of protein function due to phase separation in a particular environment.

For a particular environment, we consider α to remain the same irrespective of whether Ura3p is non‐essential, essential, or toxic (i.e., when grown in +Uracil, −Uracil, or +5FOA). In conditions where Ura3p activity is neither essential nor toxic, we consider that there is no loss or gain of function and that the differences between URA3_agg_ and URA3_sol_ are a consequence of the phase separation process. Thus, the cost of protein deposit formation due to phase transition could be expressed as:

(7) $$S=\alpha \cdot \frac{F_{\mathrm{FOCIagg}}}{F_{\mathrm{TOTALagg}}}\;\to \;\alpha =\frac{S\cdot F_{\mathrm{TOTALagg}}}{F_{\mathrm{FOCIagg}}}$$

where F_FOCIagg_ is the fluorescence contained in the deposits and F_CYTOagg_ is the florescence from the cytoplasm. The ratio quantifies the extent of phase separation. α is the magnitude of the effect of forming the deposit in specific environment.

By considering that α remains the same for a specific environment when grown in the different growth media, we can estimate the magnitude of effect for the loss (β) and gain (γ) of function when grown in the absence of uracil and in the presence of 5FOA, respectively. Therefore, when Ura3 activity is essential, the magnitude of effect of the loss of protein function due to phase separation can be expressed as:

(8) $$\begin{array}{cc} & S=\alpha \cdot \frac{F_{\mathrm{FOCIagg}}}{F_{\mathrm{TOTALagg}}}+\beta \cdot \frac{S\cdot F_{\mathrm{CYTOagg}}}{F_{\mathrm{TOTALsol}}}\;\to \\ & \beta =\left(S-\alpha \cdot \frac{F_{\mathrm{FOCIagg}}}{F_{\mathrm{TOTALagg}}}\right)\cdot \frac{F_{\mathrm{TOTALsol}}}{F_{\mathrm{CYTOagg}}} \end{array}$$

where F_TOTALsol_ is the fluorescence of the cells expressing Ura3p_sol_ measured in the corresponding environment. F_TOTALsol_ represents the 100% of active enzyme that is available to a cell in a particular environment for a non‐aggregation‐prone Ura3p_sol_. F_CYTOagg_/F_TOTALsol_ measures the amount of activity that is lost in the aggregating strain.

In conditions where Ura3 activity is toxic (i.e., uracil and 5FOA are added in the medium), the magnitude of effect of the gain of protection against the toxic activity (γ) could be calculated as below:

(9) $$\begin{array}{cc} & S=\alpha \cdot \frac{F_{FOCI_{\mathrm{agg}}}}{F_{\mathrm{TOTALagg}}}+\gamma \cdot \frac{F_{\mathrm{CYTOagg}}}{F_{\mathrm{TOTALsol}}}\;\to \\ & \gamma =\left(S-\alpha \cdot \frac{F_{\mathrm{FOCIagg}}}{F_{\mathrm{TOTALagg}}}\right)\cdot \frac{F_{\mathrm{TOTALsol}}}{F_{\mathrm{CYTOagg}}} \end{array}$$

The three effects can be integrated in one general equation that could be applied for any of the environments and Ura3p roles (essential, non‐essential, toxic) analyzed (Fig 3):

(10) $$S=\alpha \cdot \frac{F_{\mathrm{FOCIagg}}}{F_{\mathrm{TOTALagg}}}+\beta \cdot \frac{F_{\mathrm{CYTOagg}}}{F_{\mathrm{TOTALsol}}}+\gamma \cdot \frac{F_{\mathrm{CYTOagg}}}{F_{\mathrm{TOTALsol}}}$$

### mRNA expression levels

5 ml of fresh cells grown in 1% galactose was centrifuged and suspended in 1 ml of 0.2 M lithium acetate–1% SDS solution. After 5 min at 70°C, 3 ml of TRIzol^®^ was added. Next, the RNA was extracted with TRIzol^®^ Reagent following the instructions provided by Life Technologies, and the concentration of purified mRNA was measured. Retrotranscription was performed using the RevertAid H Minus First Strand cDNA Synthesis Kit (Thermo scientific) and the random hexamer primers included with the kit. The concentration of the cDNA generated was adjusted before running the qPCR experiment. The reactions were done using the SYBR^®^ Green PCR Master Mix (Life technologies) and a ViiA7 (Applied Biosystems). The pair of primers (FmRNA/RmRNA) was designed using Primer BLAST to anneal to the GFP region and not to the yeast genome (Appendix, Vectors and Primers). The mRNA of a reference gene (ALG9) was measured to normalize the data. The primers to amplify this gene were obtained from Teste *et al* (2009). Transcript quantification was done using the Δ*C*_t_ method where the variation between URA3_sol_ and URA3_agg_ was calculated as ΔΔ*C*_t_. The measured fold change between URA3_sol_ and URA3_agg_ is 1.07.

## Author contributions

MMB, NSG, and SV conceived the project. MMB and NSG designed the project and wrote the manuscript. MTB and CNJR helped with experimental design. NSG, MTB performed the experiments, analyzed the data, and performed the computational and statistical analyses. AT performed the computational simulations. NSG led the project and MMB supervised the project. All authors were involved in discussion and preparation of the final manuscript.

## Conflict of interest

The authors declare that they have no conflict of interest.

## Supporting information



Appendix

Expanded View Figures PDF

Review Process File

## Data Availability

We provide all relevant datasets in the Appendix.
