# Supplementary material for: The fitness cost and benefit of phase‐separated protein deposits
Source: Mol Syst Biol. 2019 Apr 8;15(4):e8075. doi: 10.15252/msb.20178075 (PMC6452874; doi:10.15252/msb.20178075)
Supplement: Supplementary file 1 — Appendix [file MSB-15-e8075-s001.pdf]

The fitness cost and benefit of protein phase separated protein deposits

Natalia Sanchez de Groot[1,2,3,†*], Marc Torrent Burgas[1,4†], Charles N. J. Ravarani[1], Ala Trusina[5], Salvador Ventura[6], M. Madan Babu[1,*]

[1]*MRC Laboratory of Molecular Biology, Francis Crick Avenue, Cambridge, CB2 0QH, UK*
[2]*Bioinformatics and Genomics Programme, Centre for Genomic Regulation (CRG), Dr. Aiguader 88, 08003*
[3]*Universitat Pompeu Fabra (UPF), Barcelona, Spain*
[4]*Systems Biology of Infection Lab, Department of Biochemistry and Molecular Biology, Universitat Autònoma de Barcelona, 08193, Barcelona, Spain*
[5]*Niels Bohr Institute, University of Copenhagen, Copenhagen, Denmark*
[6]*Institut de Biotecnologia i Biomedicina and Departament de Bioquímica i Biologia Molecular, Universitat Autònoma de Barcelona, 08193-Bellaterra (Barcelona), Spain*

†Both authors contributed equally

* Correspondence: *natalia.sanchez@crg.eu (N.S.G.), madanm@mrc-lmb.cam.ac.uk (M.M.B.)*

Appendix

Table of contents

Vectors and Primers 2

Appendix Table S1. List of data measured for each strain, growth media and environment. 6

Appendix Table S2. List of doubling times measured for each strain, growth media and environment. 7

Appendix Table S3. Time required to reach 50% of the population in (simulated) alternating environments. 8

Appendix Figure S1. Domain organization of proteins that can undergo phase separation and form functional deposits in vivo 11

Appendix Figure S2. Variation in fluorescence depending on the strain and the concentration of inducer. 13

Appendix Figure S3. Cell area vs. foci size distribution for $URA3_{agg}$ cells grown in different environments when Ura3p is non-essential (+URA). 17

Appendix References 19

Vectors and Primers

<u>TRP start sequence</u>

(The regions homologous to TRP1 are indicated in bold)

5′GGCGCGCCGCGGCCGCGATATC**GAGGGCATTGGTGACTATTGAGCACGTGAGTATACGTGATTAAGCACACAA**
AGGCAGCTTGGAGTCTCGAGGCGAGACGTCATCGAAGCTTACCGACTAGTACGAGAGCTCAGCCATGCATGCGAG
AATTC**AAATGGCGTTATTGGTGTTGATGTAAGCGGAGGTGTGGAGACAAATGGTGTAAAAGACTCTAACA**GCTAG
CGCCGGCGGTACC 3′

<u>Restriction map of TRP start</u>

```
                                                   SacII
                                             BssHII NotI
                                            AscI     EagI   EcoRV
    CGAATTGGCGGAAGGCCGTCAAGGCCACGTGTCTTGTCCAGGCGCGCCGCGGCCGCGATA
  1 ---------+---------+---------+---------+---------+---------+
    GCTTAACCGCCTTCCGGCAGTTCCGGTGCACAGAACAGGTCCGCGCGGCGCCGGCGCTAT

                                   AccI
    TCGAGGGCATTGGTGACTATTGAGCACGTGAGTATACGTGATTAAGCACACAAAGGCAGC
 61 ---------+---------+---------+---------+---------+---------+
    AGCTCCCGTAACCACTGATAACTCGTGCACTCATATGCACTAATTCGTGTGTTTCCGTCG
                   BsmBI                                       SphI
           XhoI      AatII     HindIII   SpeI      SacI      NsiI
    TTGGAGTCTCGAGGCGAGACGTCATCGAAGCTTACCGACTAGTACGAGAGCTCAGCCATG
121 ---------+---------+---------+---------+---------+---------+
    AACCTCAGAGCTCCGCTCTGCAGTAGCTTCGAATGGCTGATCATGCTCTCGAGTCGGTAC

           EcoRI
    CATGCGAGAATTCAAATGGCGTTATTGGTGTTGATGTAAGCGGAGGTGTGGAGACAAATG
181 ---------+---------+---------+---------+---------+---------+
    GTACGCTCTTAAGTTTACCGCAATAACCACAACTACATTCGCCTCCACACCTCTGTTTAC

                      NheI  NaeI  KpnI  PacI
    GTGTAAAAGACTCTAACAGCTAGCGCCGGCGGTACCTTAATTAATGGAGCACAAGACTGG
241 ---------+---------+---------+---------+---------+---------+
    CACATTTTCTGAGATTGTCGATCGCGGCCGCCATGGAATTAATTACCTCGTGTTCTGACC


    CCTCATGGGCCTTCCGCTCACTGC
301 ---------+---------+----
    GGAGTACCCGGAAGGCGAGTGACG
```

Fragment of TRP-URA

5' muticloning site and barcode: 1 - 130
GAL1 promoter: 131 - 595 (blue)
URA3 gene: 602 - 1402 (green)
Linker 1: 1403 - 1438
GFP gene: 1439 - 2155 (violet)
TEF terminator: 2163 - 2392 (red)
SpHis5 marker cassette: 2782 - 3668 (orange)
3' muticloning site and barcode: 3669 - 3813

5'GGCGCGCCGCGGCCGCGATATCGAGGGCATTGGTGACTATTGAGCACGTGAGTATACGTG
ATTAAGCACACAAAGGCAGCTTGGAGTCTCGAGCGTTGATGCTCTACAAGATGCCGTGCTGC
AGAAGCTTTGAAGTACGGATTAGAAGCCGCCGAGCGGGTGACAGCCCTCCGAAGGAAGACTC
TCCTCCGTGCGTCCTCGTCTTCACCGGTCGCGTTCCTGAAACGCAGATGTGCCTCGCGCCGC
ACTGCTCCGAACAATAAAGATTCTACAATACTAGCTTTTATGGTTATGAAGAGGAAAAATTG
GCAGTAACCTGGCCCCACAAACCTTCAAATGAACGAATCAAATTAACAACCATAGGATGATA
ATGCGATTAGTTTTTTAGCCTTATTTCTGGGGTAATTAATCAGCGAAGCGATGATTTTTGAT
CTATTAACAGATATATAAATGCAAAAACTGCATAACCACTTTAACTAATACTTTCAACATTT
TCGGTTTGTATTACTTCTTATTCAAATGTAATAAAAGTATCAACAAAAAATTGTTAATATAC
CTCTATACTTTAACGTCAAGGAGAAAAAACCCCGGATCCGTCGACATGTCGAAAGCTACATA
TAAGGAACGTGCTGCTACTCATCCTAGTCCTGTTGCTGCCAAGCTATTTAATATCATGCACG
AAAAGCAAACAAACTTGTGTGCTTCATTGGATGTTCGTACCACCAAGGAATTACTGGAGTTA
GTTGAAGCATTAGGTCCCAAAATTTGTTTACTAAAAACACATGTGGATATCTTGACTGATTT
TTCCATGGAGGGCACAGTTAAGCCGCTAAAGGCATTATCCGCCAAGTACAATTTTTTACTCT
TCGAAGACAGAAAATTTGCTGACATTGGTAATACAGTCAAATTGCAGTACTCTGCGGGTGTA
TACAGAATAGCAGAATGGGCAGACATTACGAATGCACACGGTGTGGTGGGCCCAGGTATTGT
TAGCGGTTTGAAGCAGGCGGCAGAAGAAGTAACAAAGGAACCTAGAGGCCTTTTGATGTTAG
CAGAATTGTCATGCAAGGGCTCCCTATCTACTGGAGAATATACTAAGGGTACTGTTGACATT
GCGAAGAGCGACAAAGATTTTGTTATCGGCTTTATTGCTCAAAGAGACATGGGTGGAAGAGA
TGAAGGTTACGATTGGTTGATTATGACACCCGGTGTGGGTTTAGATGACAAGGGAGACGCAT
TGGGTCAACAGTATAGAACCGTGGATGATGTGGTCTCTACAGGATCTGACATTATTATTGTT
GGAAGAGGACTATTTGCAAAGGGAAGGGATGCTAAGGTAGAGGGTGAACGTTACAGAAAAGC
AGGCTGGGAAGCATATTTGAGAAGATGCGGCCAGCAAAACGGTACCGCTAGTGGTTCTGCTG
GTTCTGCGATTAACATGTCTAAAGGTGAAGAATTATTCACTGGTGTTGTCCCAATTTTGGTT
GAATTAGATGGTGATGTTAATGGTCACAAATTTTCTGTCTCCGGTGAAGGTGAAGGTGATGC
TACTTACGGTAAATTGACCTTAAAATTTATTTGTACTACTGGTAAATTGCCAGTTCCATGGC
CAACCTTAGTCACTACTTTAACTTATGGTGTTCAATGTTTTTCTAGATACCCAGATCATATG
AAACAACATGACTTTTTCAAGTCTGCCATGCCAGAAGGTTATGTTCAAGAAAGAACTATTTT
TTTCAAAGATGACGGTAACTACAAGACCAGAGCTGAAGTCAAGTTTGAAGGTGATACCTTAG
TTAATAGAATCGAATTAAAAGGTATTGATTTTAAAGAAGATGGTAACATTTTAGGTCACAAA
TTGGAATACAACTATAACTCTCACAATGTTTACATCATGGCTGACAAACAAAAGAATGGTAT
CAAAGTTAACTTCAAAATTAGACACAACATTGAAGATGGTTCTGTTCAATTAGCTGACCATT
ATCAACAAAATACTCCAATTGGTGATGGTCCAGTCTTGTTACCAGACAACCATTACTTATCC
ACTCAATCTGCCTTATCCAAAGATCCAAACGAAAAGAGAGACCACATGGTCTTGTTAGAATT
TGTTACTGCTGCTGGTATTACCCATGGTATTGATGAATTGTACAAATAAGGCGCGCCACTTC
TAAATAAGCGAATTTCTTATGATTTATGATTTTTATTATTAAATAAGTTATAAAAAAAATAA
GTGTATACAAATTTTAAAGTGACTCTTAGGTTTTAAAACGAAAATTCTTATTCTTGAGTAAC

TCTTTCCTGTAGGTCAGGTTGCTTTCTCAGGTATAGTATGAGGTCGCTCTTATTGACCACAC
CTCTACCGGCAGATCCGCTAGGGATAACAGGGTAATATAGATCTGTTTAGCTTGCCTCGTCC
CCGCCGGGTCACCCGGCCAGC**GACATGGAGGCCCAGAATACCCTCCTTGACAGTCTTGACGT**
GCGCAGCTCAGGGGCATGATGTGACTGTCGCCCGTACATTTAGCCCATACATCCCCATGTAT
AATCATTTGCATCCATACATTTTGATGGCCGCACGGCGCGAAGCAAAAATTACGGCTCCTCG
CTGCAGACCTGCGAGCAGGGAAACGCTCCCCTCACAGACGCGTTGAATTGTCCCCACGCCGC
GCCCCTGTAGAGAAATATAAAAGGTTAGGATTTGCCACTGAGGTTCTTCTTTCATATACTTC
CTTTTAAAATCTTGCTAGGATACAGTTCTCACATCACATCCGAACATAAACAACCATGGGTA
GGAGGGCTTTTGTAGAAAGAAATACGAACGAAACGAAAATCAGCGTTGCCATCGCTTTGGAC
AAAGCTCCCTTACCTGAAGAGTCGAATTTTATTGATGAACTTATAACTTCCAAGCATACAAA
CCAAAAGGGAGAACAAGTAATCCAAGTAGACACGGGAATTGGATTCTTGGATCACATGTATC
ATGCACTGGCTAAACATGCAGGCTGGAGCTTACGACTTTACTCAAGAGGTGATTTAATCATC
GATGATCATCACACTGCAGAAGATACTGCTATTGCACTTGGTATTGCATTCAAGCAGGCTAT
GGAGTAACTTTGCCGGCGTTAAAAGATTTGGACATGCTTATTGTCCACTTGACGAAGCTCTT
TCTAGAAGCGTAGTTGACTTGTCGGGACGGCCCTATGCTGTTATCGATTTGGGATTAAAGCG
TGAAAAGGTTGGGGAATTGTCCTGTGAAATGATCCCTCACTTACTATATTCCTTTTCGGTAG
CAGCTGGAATTACTTTGCATGTTACCTGCTTATATGGTAGTAATGACCATCATCGTGCTGAA
AGCGCTTTTAAATCTCTGGCTGTTGCCATGCGCGGCTACTAGTCTTACTGGAAGTTCTGA
AGTCCCAAGCACGAAGGGAGTGTTGTAAAGAGTACTGACAATAAAAAGATTCTTGTTTTCAA
GAACTTGTCATTTGTATAGTTTTTTTATATTGTAGTTGTTCTATTTTAATCAAATGTTAGCG
TGATTTATATTTTTTTTCGCCTCGACATCATCTGCCCAGATGCGAAGTTAAGTGCGCAGAAA
GTAATATCATGCGTCAATCGTATGTGAATGCTGGTCGCTATACTGCTGTCGATTCGATACTA
ACGCCGCCATCCAGTTTAAACGAGCTCCATGTGGTTGCCTTACGCCGACTACGGATCCATGC
ATGCGAGAATTCAAATGGCGTTATTGGTGTTGATGTAAGCGGAGGTGTGGAGACAAATGGTG
TAAAAGACTCTAACAGCTAGCGCCGGCGGTACC 3'

Fragment of pMA vector (residues 3814 – 6183 from TRP-URA vector):

5'ttaattaatggagcacaagactggcctcatgggccttccgctcactgcccgctttccagt
cgggaaacctgtcgtgccagctgcattaacatggtcatagctgtttccttgcgtattgggcg
ctctccgcttcctcgctcactgactcgctgcgctcggtcgttcgggtaaagcctggggtgcc
taatgagcaaaaggccagcaaaaggccaggaaccgtaaaaaggccgcgttgctggcgttttt
ccataggctccgcccccctgacgagcatcacaaaaatcgacgctcaagtcagaggtggcgaa
acccgacaggactataaagataccaggcgtttccccctggaagctccctcgtgcgctctcct
gttccgaccctgccgcttaccggatacctgtccgcctttctcccttcgggaagcgtggcgct
ttctcatagctcacgctgtaggtatctcagttcggtgtaggtcgttcgctccaagctgggct
gtgtgcacgaaccccccgttcagcccgaccgctgcgccttatccggtaactatcgtcttgag
tccaacccggtaagacacgacttatcgccactggcagcagccactggtaacaggattagcag
agcgaggtatgtaggcggtgctacagagttcttgaagtggtggcctaactacggctacacta
gaagaacagtatttggtatctgcgctctgctgaagccagttaccttcggaaaaagagttggt
agctcttgatccggcaaacaaaccaccgctggtagcggtggtttttttgtttgcaagcagca
gattacgcgcagaaaaaaaggatctcaagaagatcctttgatcttttctacggggtctgacg
ctcagtggaacgaaaactcacgttaagggattttggtcatgagattatcaaaaaggatcttc
acctagatccttttaaattaaaaatgaagttttaaatcaatctaaagtatatatgagtaaac
ttggtctgacagttaccaatgcttaatcagtgaggcacctatctcagcgatctgtctatttc
gttcatccatagttgcctgactccccgtcgtgtagataactacgatacgggagggcttacca
tctggccccagtgctgcaatgataccgcgagaaccacgctcaccggctccagatttatcagc
aataaaccagccagccggaagggccgagcgcagaagtggtcctgcaactttatccgcctcca
tccagtctattaattgttgccgggaagctagagtaagtagttcgccagttaatagtttgcgc

aacgttgttgccattgctacaggcatcgtggtgtcacgctcgtcgtttggtatggcttcatt
cagctccggttcccaacgatcaaggcgagttacatgatcccccatgttgtgcaaaaaagcgg
ttagctccttcggtcctccgatcgttgtcagaagtaagttggccgcagtgttatcactcatg
gttatggcagcactgcataattctcttactgtcatgccatccgtaagatgcttttctgtgac
tggtgagtactcaaccaagtcattctgagaatagtgtatgcggcgaccgagttgctcttgcc
cggcgtcaatacgggataataccgcgccacatagcagaactttaaaagtgctcatcattgga
aaacgttcttcggggcgaaaactctcaaggatcttaccgctgttgagatccagttcgatgta
acccactcgtgcacccaactgatcttcagcatcttttactttcaccagcgtttctgggtgag
caaaaacaggaaggcaaaatgccgcaaaaaagggaataagggcgacacggaaatgttgaata
ctcatactcttcctttttcaatattattgaagcatttatcagggttattgtctcatgagcgg
atacatatttgaatgtatttagaaaaataaacaaataggggttccgcgcacatttccccgaa
aagtgccacctaaattgtaagcgttaatattttgttaaaattcgcgttaaatttttgttaaa
tcagctcattttttaaccaataggccgaaatcggcaaaatcccttataaatcaaaagaatag
accgagatagggttgagtggccgctacagggcgctcccattcgccattcaggctgcgcaact
gttgggaagggcgtttcggtgcgggcctcttcgctattacgccagctggcgaaagggggatg
tgctgcaaggcgattaagttgggtaacgccagggttttcccagtcacgacgttgtaaaacga
cggccagtgagcgcgacgtaatacgactcactatagggcgaattggcggaaggccgtcaagg
ccacgtgtcttgtcca 3'

Fragment of TRP-URA-AB

Linker of 12 amino acids (GGSANGTSGASG) (Supplemental Experimental Procedures) and fragment of Aβ was added between GFP and the TEF terminator. Insertion site after residue 2152 of TRP-URA vector (uppercase Linker 2 and bold Aβ42 sequence):

5'GGTGGAAGTGCTAATGGTACTTCTGGTGCTAGTGGT**gatgctgaatttagacatgattct**
ggttatgaagttcatcatcaaaaattggtctttttgctgaagatgttggttctaataaagg
tgctattattggtttgatggttggtggtgttgttattgct 3'

Sequences of the primers used

Primers for qPCR, to monitor the competition assay:
solF: TCCAAACGAAAAGAGAGACCACA
solR: AGTGGCGCGCCTTATTTGTA

aggF: TGGTGCTAGTGGTGATGCTG
aggR: AGAACCAACATCTTCAGCAAAAA

Primers to measure the production of transcripts:
FmRNA: TAGTCACTACTTTAACTTATGGTGTTCAA
RmRNA: CTTTCTTGAACATAACCTTCTGGC

Appendix Table S1. List of data measured for each strain, growth media and environment.

Media	Envt.	S[a]	SEM	FOCI rate[b]	$F_{FOCIagg}$[c]	$F_{CYTOagg}$[d]	$F_{TOTALagg}$[e]	$F_{TOTALsol}$[f]	$\frac{F_{FOCIagg}}{F_{TOTALagg}}$	$\frac{F_{CYTOagg}}{F_{TOTALsol}}$	Coeff.[g]
5FOA	Sorbitol	0.013	±6.29E-04	0.708	5042	9613	14655	51114	0.344	0.188	0.091
	Proline	0.007	±2.82E-04	0.478	1703	7377	9080	27854	0.188	0.265	0.027
	NaCl	0.006	±2.08E-04	0.308	1917	5791	7708	31935	0.249	0.181	0.019
	H_2O_2	0.028	±1.38E-03	0.681	2869	5567	8436	33762	0.340	0.165	0.156
	DTT	0.022	±1.01E-03	0.705	3989	5183	9172	31855	0.435	0.163	0.151
	37°C	-	-	0.63	-	1411	1704	18688	-	0.075	-
	30°C	0.055	±2.71E-03	0.584	2893	5854	8746	31595	0.331	0.185	0.285
	25°C	0.002	±6.46E-05	0.884	5370	4766	10136	32962	0.530	0.145	0.061
-URA	Sorbitol	-0.003	±-6.14E-05	0.676	2445	5584	8029	27919	0.305	0.2	-0.013
	Proline	-0.004	±-2.08E-04	0.579	1186	4487	5673	9850	0.209	0.456	-0.009
	NaCl	-0.004	±-1.76E-04	0.297	606	3398	4005	23828	0.151	0.143	-0.029
	H_2O_2	-0.010	±-5.23E-04	0.247	477	3471	3949	24096	0.121	0.144	-0.073
	DTT	-0.007	±-2.86E-04	0.197	490	3296	3786	17186	0.129	0.192	-0.034
	37°C	-0.103	±-5.08E-03	0.689	2660	2990	5650	11737	0.471	0.071	-0.403
	30°C	-0.045	±-2.19E-03	0.477	691	3183	3874	18281	0.178	0.174	-0.258
	25°C	-0.01	±-4.65E-04	0.301	1107	4893	6001	16015	0.184	0.306	-0.031
+URA	Sorbitol	-0.005	±-2.36E-04	0.865	5773	6559	12332	44318	0.468	0.148	-0.011
	Proline	-0.001	±4.26E-05	0.471	1323	4491	5814	24718	0.228	0.182	-0.002
	NaCl	0.003	±8.17E-05	0.707	2413	6462	8874	38575	0.272	0.168	0.01
	H_2O_2	0.002	±2.77E-05	0.71	2961	5291	8252	42580	0.359	0.124	0.007
	DTT	-0.001	±-1.63E-05	0.321	579	3022	3601	31586	0.161	0.096	-0.007
	37°C	0.001	±2.56E-05	0.578	1360	2885	4245	9008	0.320	0.091	0.002
	30°C	0.002	±7.36E-05	0.733	2853	5908	8762	29966	0.326	0.197	0.006
	25°C	-0.005	±-1.38E-04	0.886	4209	7049	11258	34907	0.374	0.202	-0.012

a. Selection coefficient is calculated as $S = \frac{1}{\log_2 e}\left(\varpi_{agg} - \varpi_{sol}\right)$.

b. Ratio of cells with foci in that population.

c. Average fluorescence intensity emitted by a foci in the $URA3_{agg}$ population. This is calculated as: $FOCIagg \cdot FOCI_{rate}$

where FOCIagg is the average fluorescence intensity emitted by a foci taking into account only the fraction of $URA3_{agg}$ population containing foci, and the $FOCI_{rate}$ is the ratio of cells containing foci.

d. Average fluorescence intensity emitted by the cytoplasm of the $URA3_{agg}$ population. This is calculated as: $F_{CYTOagg} = F_{TOTALagg} - F_{FOCIagg}$

e. $F_{TOTALagg}$ is the average of the total fluorescence emitted by $URA3_{agg}$ population.

f. Average fluorescence intensity emitted by the $URA3_{sol}$ population.

g. Coefficient of proportionality (i.e. magnitude of effect) defined by the growth conditions. It indicates how the growth conditions influences the magnitude of a particular effect (α, for deposit formation (+URA); β for loss of function (-URA); γ for gain of protective effect (+5FOA)).

Appendix Table S2. List of doubling times measured for each strain, growth media and environment.

	-URA (essential)		+URA (non-essential)		+5FOA (toxic)	
	URA3agg	URA3sol	URA3agg	URA3sol	URA3agg	URA3sol
30ºC	3.42±0.65	2.91±0.13	2.85±0.08	2.77±0.16	4.96±0.20	5.20±0.62
Proline	2.95±0.01	2.90±0.04	2.89±0.03	2.86±0.02	4.84±0.11	3.86±0.01
NaCl	3.24±0.05	3.17±0.02	3.15±0.01	3.12±0.09	5.39±0.20	4.76±0.07
Sorbitol	4.34±0.19	4.51±0.06	4.14±0.16	4.13±0.08	9.15±1.61	6.55±0.46
H_2O_2	3.42±0.09	2.70±0.00	2.79±0.02	2.78±0.05	5.04±0.00	5.48±0.31
DTT	2.93±0.12	2.87±0.01	2.78±0.02	2.83±0.03	5.62±0.17	5.20±0.04
37ºC	-	6.26±0.01	2.68±0.03	2.64±0.01	3.02±0.16	7.21±0.66
25ºC	4.43±0.14	4.00±0.21	4.21±0.22	4.03±0.21	6.06±0.11	6.69±0.00

Doubling times calculated from the growth curve obtained by following the turbidity (OD600nm) and fluorescence (450 nm excitation & 510 nm emission) during 72 hours with a Tecan Infinite M200 Pro. Here we show the average doubling time value from two independent experiments. Time measured in hours.

Appendix Table S3. Time required to reach 50% of the population in (simulated) alternating environments.

Environment 1		Environment 2		Selection coefficient of the history of environments (S_h)	Time (in generations) to reach 50% of the population
H2O2	FOA	30	FOA	0.0415	126
DTT	FOA	30	FOA	0.0385	136
Sorbitol	FOA	30	FOA	0.0340	154
Proline	FOA	30	FOA	0.0310	169
NaCl	FOA	30	FOA	0.0305	171
30	FOA	NaCl	URA+	0.0290	179
30	FOA	25	FOA	0.0285	185
30	FOA	H2O2	URA+	0.0285	185
30	FOA	30	URA+	0.0285	185
30	FOA	37	URA+	0.0280	187
30	FOA	DTT	URA+	0.0270	195
30	FOA	Proline	URA+	0.0270	195
30	FOA	Sorbitol	URA-	0.0260	203
30	FOA	Proline	URA-	0.0255	205
30	FOA	NaCl	URA-	0.0255	205
30	FOA	Sorbitol	URA+	0.0250	212
30	FOA	25	URA+	0.0250	212
H2O2	FOA	DTT	FOA	0.0250	209
30	FOA	DTT	URA-	0.0240	216
30	FOA	H2O2	URA-	0.0225	232
30	FOA	25	URA-	0.0225	232
Sorbitol	FOA	H2O2	FOA	0.0205	255
Proline	FOA	H2O2	FOA	0.0175	299
Sorbitol	FOA	DTT	FOA	0.0175	298
NaCl	FOA	H2O2	FOA	0.0170	308
H2O2	FOA	NaCl	URA+	0.0155	339
H2O2	FOA	25	FOA	0.0150	350
H2O2	FOA	H2O2	URA+	0.0150	350
H2O2	FOA	30	URA+	0.0150	349
Proline	FOA	DTT	FOA	0.0145	360
H2O2	FOA	37	URA+	0.0145	360
NaCl	FOA	DTT	FOA	0.0140	375
H2O2	FOA	DTT	URA+	0.0135	387
H2O2	FOA	Proline	URA+	0.0135	387
DTT	FOA	NaCl	URA+	0.0125	420
H2O2	FOA	Sorbitol	URA-	0.0125	416
H2O2	FOA	Proline	URA-	0.0120	439
H2O2	FOA	NaCl	URA-	0.0120	439
DTT	FOA	H2O2	URA+	0.0120	437

DTT	FOA	25	FOA	0.0120	436
DTT	FOA	30	URA+	0.0120	436
DTT	FOA	37	URA+	0.0115	457
H2O2	FOA	Sorbitol	URA+	0.0115	455
H2O2	FOA	25	URA+	0.0115	455
DTT	FOA	DTT	URA+	0.0105	497
DTT	FOA	Proline	URA+	0.0105	497
H2O2	FOA	DTT	URA-	0.0105	496
Sorbitol	FOA	Proline	FOA	0.0100	520
DTT	FOA	Sorbitol	URA-	0.0095	550
Sorbitol	FOA	NaCl	FOA	0.0095	547
DTT	FOA	Proline	URA-	0.0090	585
DTT	FOA	NaCl	URA-	0.0090	585
H2O2	FOA	H2O2	URA-	0.0090	579
H2O2	FOA	25	URA-	0.0090	579
DTT	FOA	Sorbitol	URA+	0.0085	614
DTT	FOA	25	URA+	0.0085	614
Sorbitol	FOA	NaCl	URA+	0.0080	649
DTT	FOA	DTT	URA-	0.0075	696
Sorbitol	FOA	H2O2	URA+	0.0075	693
Sorbitol	FOA	25	FOA	0.0075	692
Sorbitol	FOA	30	URA+	0.0075	691
Sorbitol	FOA	37	URA+	0.0070	740
Proline	FOA	NaCl	FOA	0.0065	805
DTT	FOA	H2O2	URA-	0.0060	873
DTT	FOA	25	URA-	0.0060	869
Sorbitol	FOA	DTT	URA+	0.0060	867
Sorbitol	FOA	Proline	URA+	0.0060	860
Proline	FOA	NaCl	URA+	0.0050	1046
30	FOA	30	URA-	0.0050	1029
Sorbitol	FOA	Sorbitol	URA-	0.0050	1024
NaCl	FOA	NaCl	URA+	0.0045	1164
Proline	FOA	H2O2	URA+	0.0045	1163
Proline	FOA	25	FOA	0.0045	1162
Proline	FOA	30	URA+	0.0045	1161
Sorbitol	FOA	Proline	URA-	0.0045	1150
Sorbitol	FOA	NaCl	URA-	0.0045	1150
NaCl	FOA	H2O2	URA+	0.0040	1311
NaCl	FOA	25	FOA	0.0040	1309
NaCl	FOA	30	URA+	0.0040	1308
Proline	FOA	37	URA+	0.0040	1305
Sorbitol	FOA	Sorbitol	URA+	0.0040	1283
Sorbitol	FOA	25	URA+	0.0040	1279

NaCl	FOA	37	URA+	0.0035	1494
Proline	FOA	DTT	URA+	0.0030	1752
Proline	FOA	Proline	URA+	0.0030	1734
Sorbitol	FOA	DTT	URA-	0.0030	1693
NaCl	FOA	DTT	URA+	0.0025	2113
NaCl	URA+	H2O2	URA+	0.0025	2100
25	FOA	NaCl	URA+	0.0025	2095
NaCl	URA+	30	URA+	0.0025	2093
NaCl	FOA	Proline	URA+	0.0025	2086
25	FOA	H2O2	URA+	0.0020	2625
H2O2	URA+	30	URA+	0.0020	2622
25	FOA	30	URA+	0.0020	2614
NaCl	URA+	37	URA+	0.0020	2612
Proline	FOA	Sorbitol	URA-	0.0020	2578
Proline	FOA	Proline	URA-	0.0015	3537
Proline	FOA	NaCl	URA-	0.0015	3529
H2O2	URA+	37	URA+	0.0015	3492
25	FOA	37	URA+	0.0015	3477
37	URA+	30	URA+	0.0015	3472
NaCl	FOA	Sorbitol	URA-	0.0015	3436
Sorbitol	FOA	H2O2	URA-	0.0015	3333
Sorbitol	FOA	25	URA-	0.0015	3320
NaCl	FOA	Proline	URA-	0.0010	5365
NaCl	FOA	NaCl	URA-	0.0010	5360
NaCl	URA+	DTT	URA+	0.0010	5351
Proline	FOA	Sorbitol	URA+	0.0010	5197
Proline	URA+	NaCl	URA+	0.0010	5188
Proline	FOA	25	URA+	0.0010	5133
H2O2	URA+	DTT	URA+	0.0005	11065
25	FOA	DTT	URA+	0.0005	10928
DTT	URA+	30	URA+	0.0005	10874
NaCl	FOA	Sorbitol	URA+	0.0005	10443
Proline	URA+	H2O2	URA+	0.0005	10400
25	FOA	Proline	URA+	0.0005	10274
Proline	URA+	30	URA+	0.0005	10228
NaCl	FOA	25	URA+	0.0005	10188

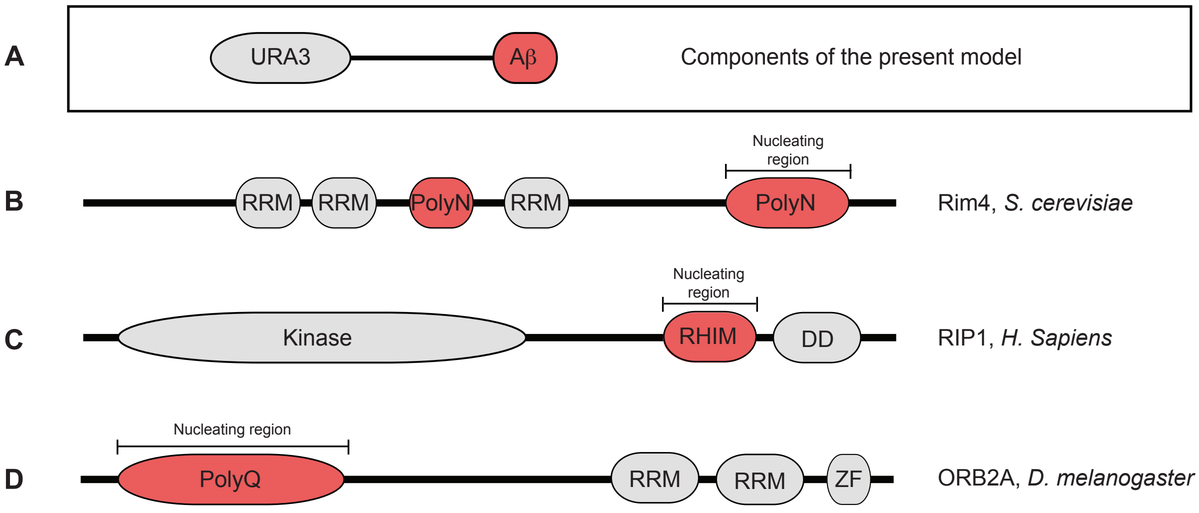

Appendix Figure S1. Domain organization of proteins that can undergo phase separation and form functional deposits *in vivo*.

A. The protein model for the current work has been designed to have a domain with a defined biochemical activity in the cell (enzymatic domain; URA3; grey) and a region that drives phase separation by simple coacervation (Aβ). Nature has selected for proteins with functions and properties separated into specific domains/regions. Here we present three natural proteins that phase separates by simple coacervation and contain spatially separated functional domains.

B. RIM4 represses several mRNAs capturing them in intracellular deposits. Rim4 aggregation is driven by a domain rich in Asparagines (polyN) and is independent of the presence of RNA presence (Berchowitz et al, 2015).

C. RIP1 and RIP3 are kinases that interact through a common region, the RIP homotypic interaction motif (RHIM). RHIM region drives the formation of amyloid deposits. *In vivo* RIP1 and RIP3 form a heterodimeric amyloid structure that mediates programmed necrosis (Li et al, 2012).

D. ORB2A is an isoform of ORB2, an orthologue of CPEB. ORB2A is crucial for the formation of ORB2 amyloid oligomers and the persistence of long-term memory (Khan et al, 2015).

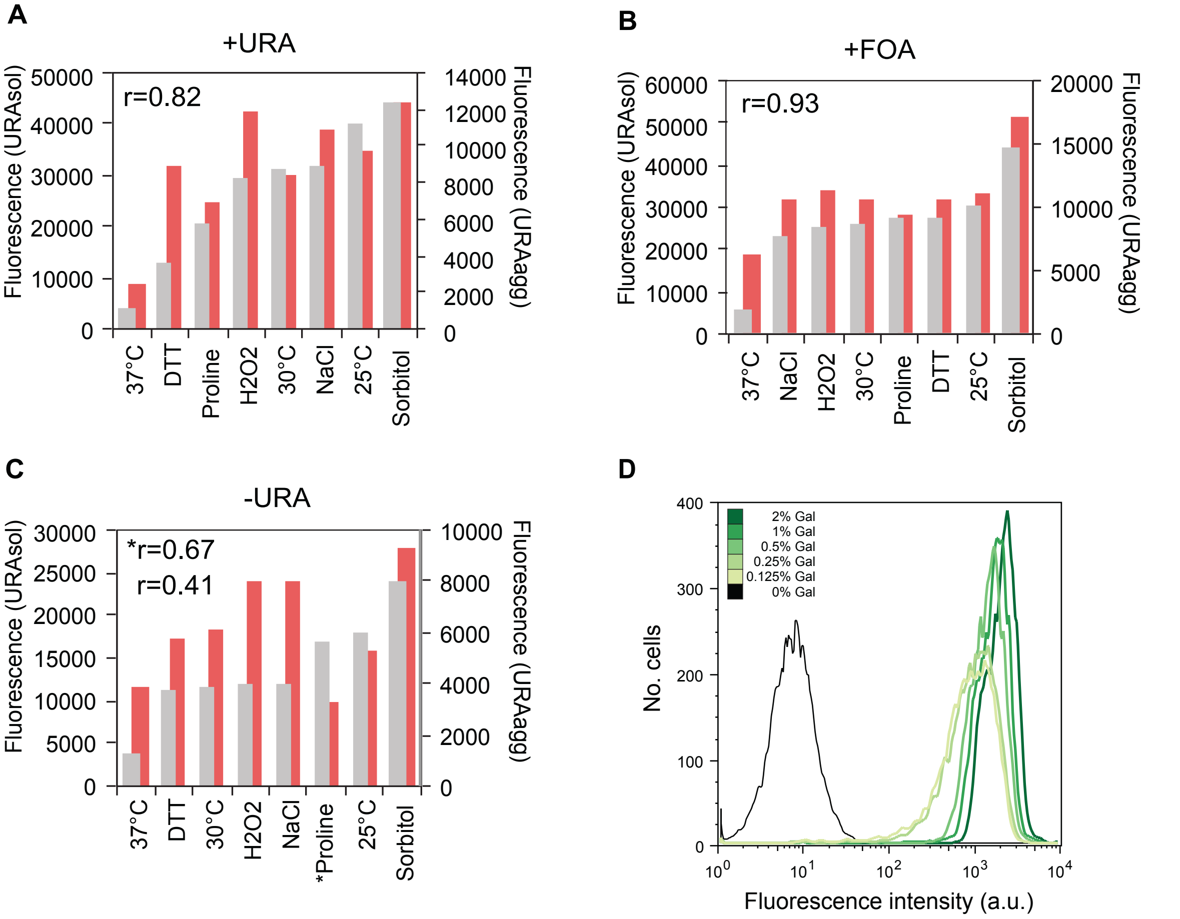

Appendix Figure S2. Variation in fluorescence depending on the strain and the concentration of inducer. Fluorescence average (arbitrary units) of the $URA3_{agg}$ (red, $F_{TOTALsol}$) and $URA3_{sol}$ (grey, $F_{TOTALagg}$) populations grown at 30°C and in media with **A**, uracil, **B**, 5FOA or **C**, without uracil. Pearson's correlation coefficient, *r*, is shown in each panel. Data is arranged from smaller to larger $URA3_{agg}$ fluorescence values for each media composition. *Correlation coefficient obtained without the Proline environmental condition. Note: Although both strains ($URA3_{agg}$ and $URA3_{sol}$) present similar transcript levels (**Figure EV2**), due to deposit formation, a part of the translated $Ura3p_{agg}$ can be rapidly removed through autophagy or other mechanisms (Sanchez de Groot et al, 2015; Villar-Pique & Ventura, 2013) and hence may lead to a reduction in $F_{TOTALagg}$ with respect to $F_{TOTALsol}$ (Sanchez de Groot et al, 2015; Villar-Pique & Ventura, 2013) (**Methods**).

D. Distribution of $URA3_{sol}$ cells in terms of their total fluorescence after 20h of growth in different galactose concentrations. Each sample was centrifuged and suspended in PBS. The samples were vortexed for 1 min before measuring the fluorescence loss using a BD LSR II flow cytometer system (BD Biosciences). 10000 cells were counted at a maximum flow rate of 600 events per second. GFP fluorescence was measured using a 488 nm laser for excitation and a 525/50 nm band pass filter.

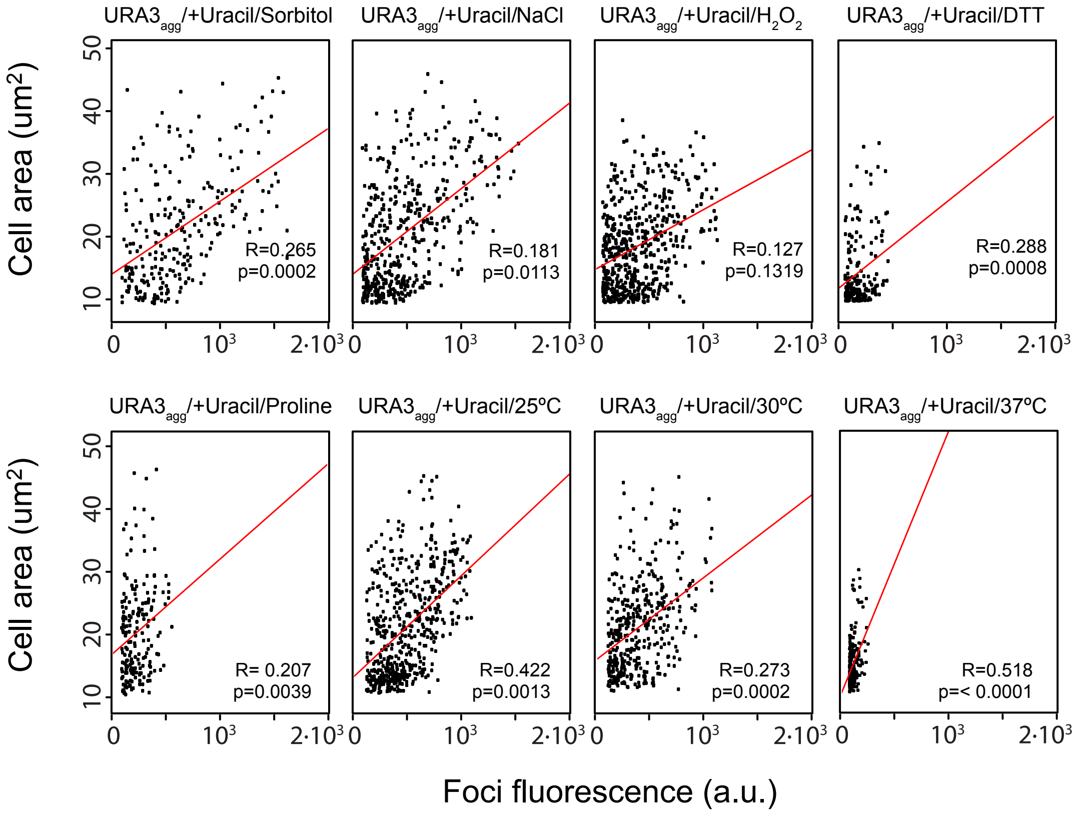

Appendix Figure S3. Cell area vs. foci size distribution for URA3$_{agg}$ cells grown in different environments when Ura3p is non-essential (+URA).

Scatter plots showing the relationship between the foci size and the cell area of the URA3agg population in a medium containing uracil (+URA). The environments are shown above. R denotes the Pearson's correlation coefficient. P-values for the correlation coefficient were measured using the R statistical package.

Appendix References

Berchowitz LE, Kabachinski G, Walker MR, Carlile TM, Gilbert WV, Schwartz TU, Amon A (2015) Regulated Formation of an Amyloid-like Translational Repressor Governs Gametogenesis. *Cell* **163:** 406-418

Kaganovich D, Kopito R, Frydman J (2008) Misfolded proteins partition between two distinct quality control compartments. *Nature* **454:** 1088-1095

Khan MR, Li L, Perez-Sanchez C, Saraf A, Florens L, Slaughter BD, Unruh JR, Si K (2015) Amyloidogenic Oligomerization Transforms Drosophila Orb2 from a Translation Repressor to an Activator. *Cell* **163:** 1468-1483

Li J, McQuade T, Siemer AB, Napetschnig J, Moriwaki K, Hsiao YS, Damko E, Moquin D, Walz T, McDermott A, Chan FK, Wu H (2012) The RIP1/RIP3 necrosome forms a functional amyloid signaling complex required for programmed necrosis. *Cell* **150:** 339-350

Sanchez de Groot N, Gomes RA, Villar-Pique A, Babu MM, Coelho AV, Ventura S (2015) Proteome response at the edge of protein aggregation. *Open Biol* **5:** 140221

Teste MA, Duquenne M, Francois JM, Parrou JL (2009) Validation of reference genes for quantitative expression analysis by real-time RT-PCR in Saccharomyces cerevisiae. *BMC Mol Biol* **10:** 99

Villar-Pique A, Ventura S (2013) Protein aggregation propensity is a crucial determinant of intracellular inclusion formation and quality control degradation. *Biochim Biophys Acta* **1833:** 2714-2724
