# Supplementary material for: The fitness cost and benefit of phase‐separated protein deposits
Source: Mol Syst Biol. 2019 Apr 8;15(4):e8075. doi: 10.15252/msb.20178075 (PMC6452874; doi:10.15252/msb.20178075)
Supplement: Supplementary file 2 — Expanded View Figures PDF [file MSB-15-e8075-s002.pdf]

Expanded View Figures

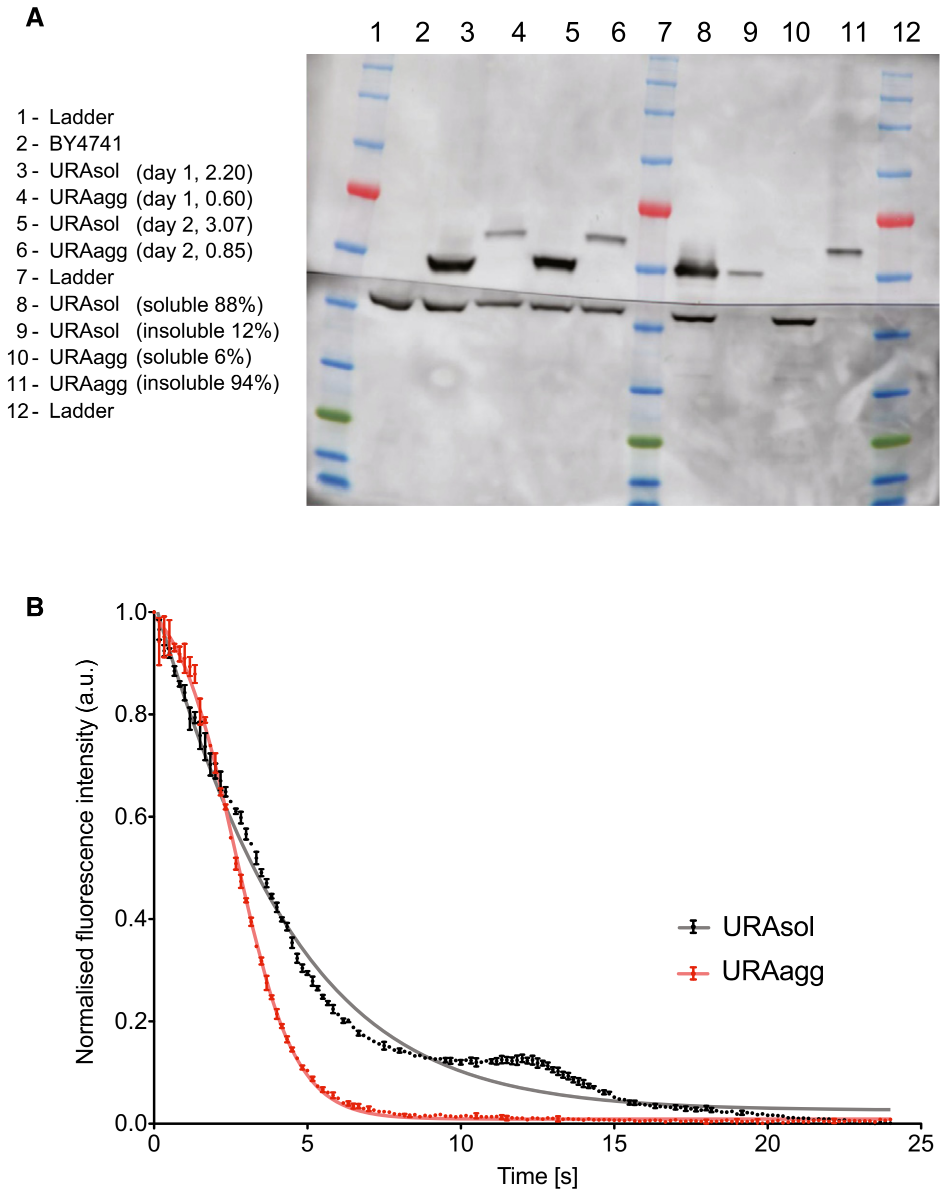

	URAagg	URAsol
**Slope**	3.456	0.9729
**Std. Error**	0.1192	0.0084
R^2	0.9881	0.9987

Figure EV1. Protein levels and half-life of URA3$_{sol}$ and URA3$_{agg}$.

A Analysis of the expression and the soluble/insoluble part of URA3$_{sol}$ and URA3$_{agg}$. Total protein fraction obtained by two independent experiments after 18 h of induction. The number indicates the Ura3p concentration (upper bands) quantification normalized by the concentration of PGK1p (lower bands). URA3$_{sol}$ distribution is 88% soluble and 12% insoluble. URA3$_{agg}$ is 6% soluble and 94% insoluble. Band analysis has been done with ImageJ.

B *In vivo* degradation rate was measured as the fluorescence loss after induction inhibition (see the Materials and Methods section). The plot shows the fluorescence normalized by the concentration of cells (absorbance at 600 nm) at each time point. The slope (absolute value), standard error, and R^2 of three technical replicates obtained from two different experiments are shown. The data were fitted to a Boltzmann's sigmoid.

© 2019 MRC Laboratory of Molecular Biology

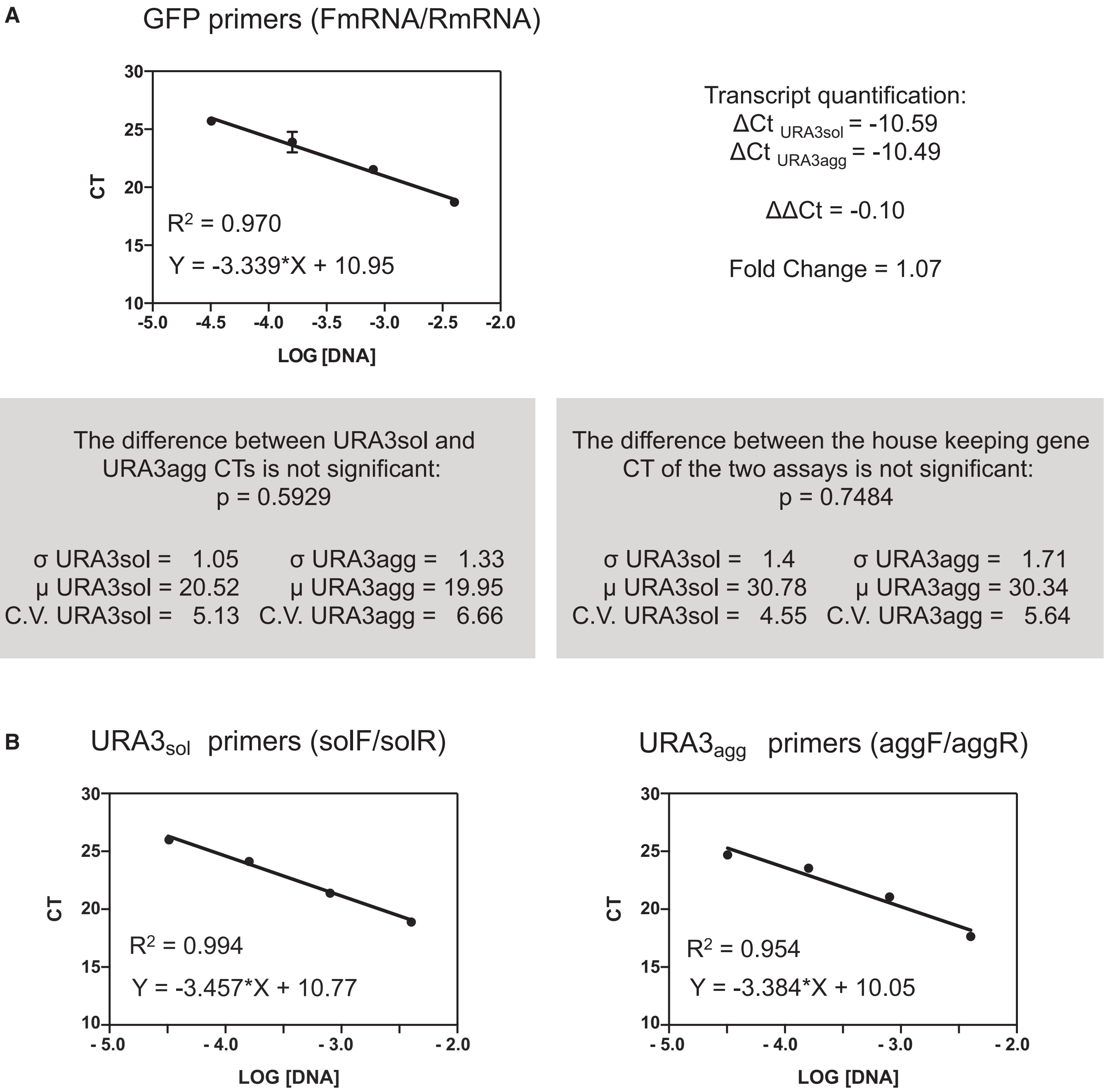

Figure EV2. Variation in transcript expression and PCR efficiency.

A URA3$_{sol}$ and URA3$_{agg}$ have similar mRNA abundance according to the $\Delta\Delta C_t$ method (Teste *et al*, 2009). These measurements were made with a pair of primers that anneal to a common region located in the GFP sequence, named primers FmRNA and RmRNA, respectively (Materials and Methods). The standard curves obtained for these primers are shown.

B Standard curves obtained for the population quantification (solF/solR primers for URA3$_{sol}$ cells and the aggF/aggR primers for URA3$_{agg}$) (Materials and Methods).

© 2019 MRC Laboratory of Molecular Biology

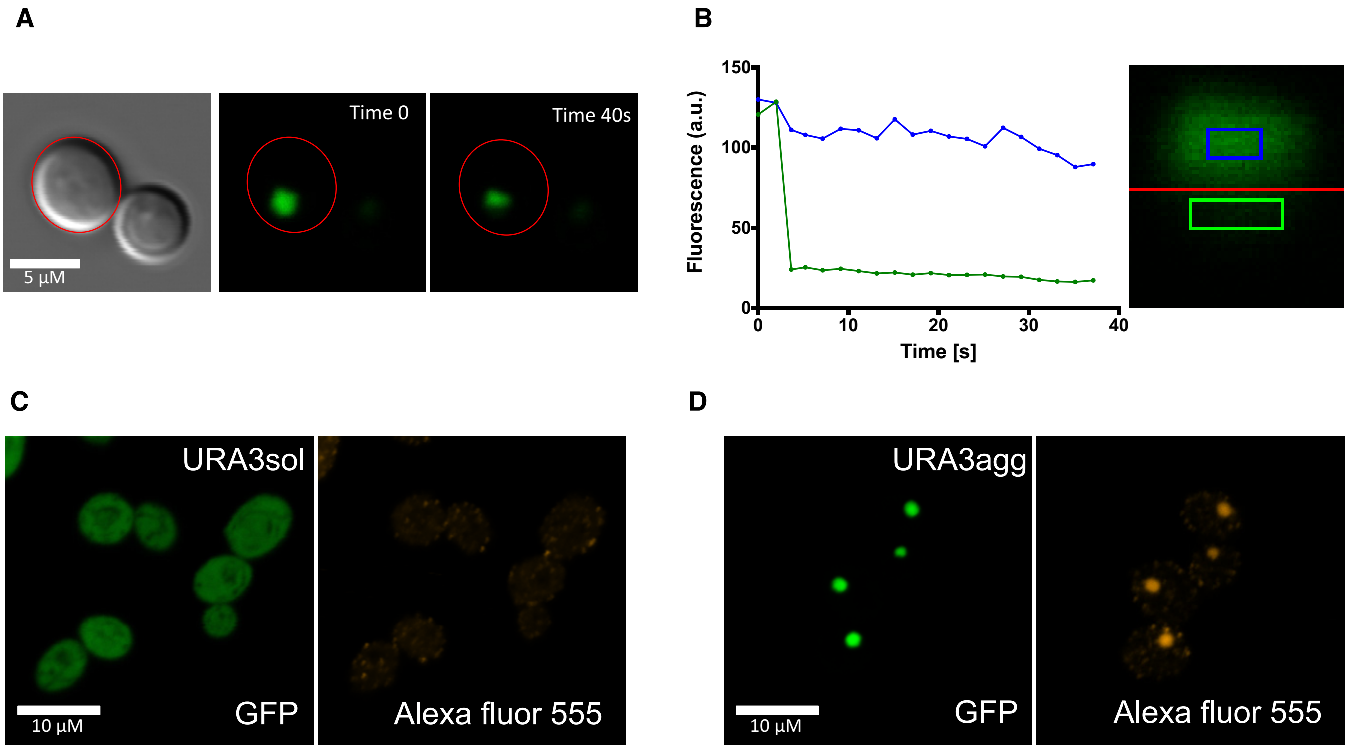

Figure EV3. Properties of URA3$_{agg}$ deposits using FRAP.

We tested the consistency of the URA3$_{agg}$ foci by photobleaching one half of the deposit (green line and green square) and monitoring the protein diffusion from the other half (blue line and blue square) with confocal microscopy. The assay was monitored for 40 seconds and shows no fluorescence loss or gain in any of the sides. This indicates that these foci are very dense, very much like an insoluble protein deposit (IPOD; Kaganovich *et al*, 2008).

A Foci before (Time 0 s) and after (Time 40 s) photobleaching.

B Graph showing the fluorescence changes along time in each side of the foci.

C, D We also tested the conformation of the protein enclosed in the URA3$_{agg}$ foci by analyzing the binding of anti-oligomer antibodies (rabbit anti-oligomer (A11) AHB0052). As a secondary antibody, we used goat anti-rabbit IgG H&L tagged with an Alexa Fluor® 555 (ab150078). We obtained a bright fluorescence signal that colocalizes with the foci's GFP fluorescence, indicating that the deposits are rich in oligomeric structures. As a control, we also incubated URA3$_{sol}$ with these antibodies obtaining few faint Alexa Fluor foci at the cytoplasm, indicating the absence or very low presence of oligomers.

© 2019 MRC Laboratory of Molecular Biology

A

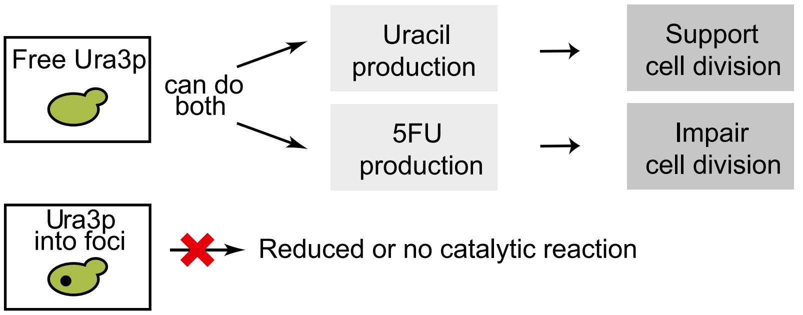

B

ENVIRONMENT	ENVIRONMENT EFFECT	URA3p ACTIVITY	CELLULAR DEMAND	SELECTION OUTCOME	
Uracil in the media (+URA)	URA3p activity is not essential	Neutral	No demand	URA3sol ≈ URA3agg	Minor effects
Favours folding (25ºC,Pro, Sorbitol, NaCl)	Abundant free & active URA3p	Toxic<br>Essential	Demand met	URA3sol ≈ URA3agg<br>URA3sol ≈ URA3agg	Minor effects
Impairs folding (37ºC, H_2O_2,DTT)	Scarce free & active URA3p	Toxic<br>Essential	Demand not met	URA3sol << URA3agg<br>URA3sol >> URA3agg	Major effects
Optimal growth (30ºC)	Fast cell division faster uracil consumption	Toxic<br>Essential	Demand not met	URA3sol << URA3agg<br>URA3sol >> URA3agg	Major effects

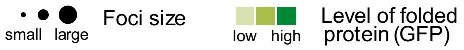

Figure EV4. Summary of the effects associated with URA3$_{agg}$ phase separation in different environments that affect the topology of Ura3p, and hence protein activity (supply) as well as the cell state (demand).

A Diagram showing different activity associated with the free and deposited protein.

B Summary table of the effects associated with Ura3p$_{agg}$ deposition and their effects on the selection outcome when growing in competition with URA3$_{sol}$.

© 2019 MRC Laboratory of Molecular Biology

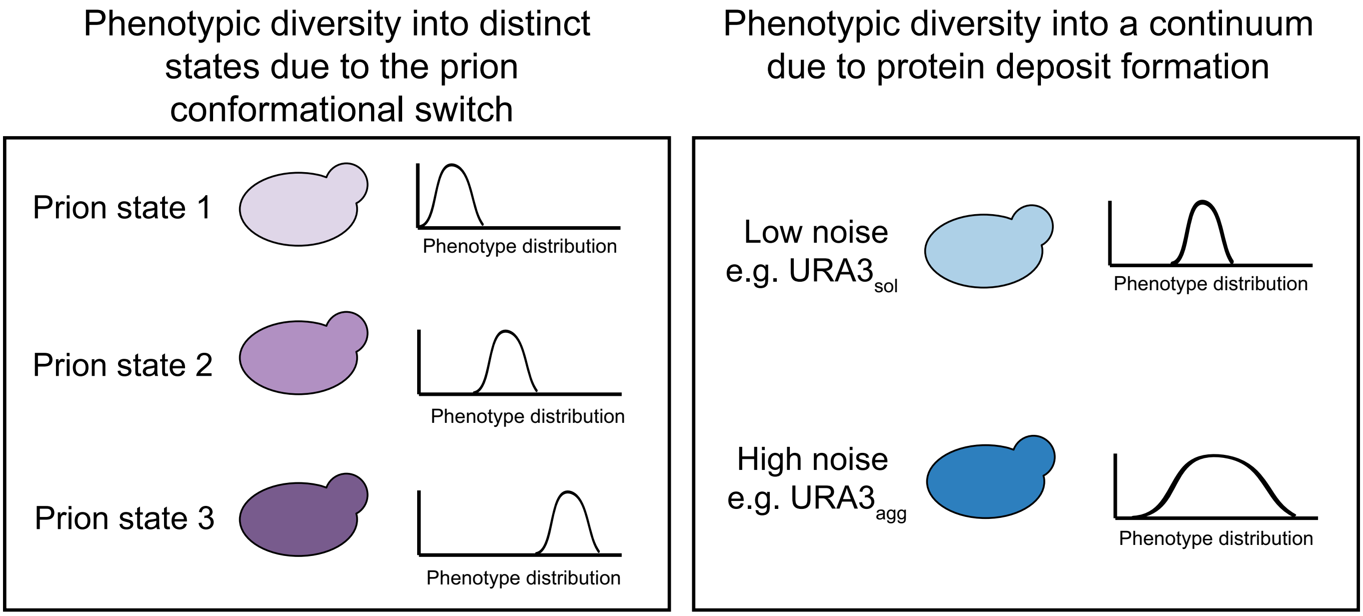

Figure EV5. Population diversity introduced by prion conformational switching and by deposit formation.

Prion conformational switching is associated with the emergence of different prion strains that provide new heritable phenotypes with distinct and largely non-overlapping phenotypes or functional states, whereas, a more general protein deposit formation upon phase separation may create a phenotypic continuum.

© 2019 MRC Laboratory of Molecular Biology
